# Identification of non-conserved residues essential for improving the hydrocarbon-producing activity of cyanobacterial aldehyde-deformylating oxygenase

**DOI:** 10.1186/s13068-019-1409-8

**Published:** 2019-04-17

**Authors:** Hisashi Kudo, Yuuki Hayashi, Munehito Arai

**Affiliations:** 1 Department of Life Sciences, Graduate School of Arts and Sciences, The University of Tokyo, 3-8-1 Komaba, Meguro, Tokyo, 153-8902 Japan; 2 Department of Physics, Graduate School of Science, The University of Tokyo, 3-8-1 Komaba, Meguro, Tokyo, 153-8902 Japan

**Keywords:** Aldehyde-deformylating oxygenase, Biohydrocarbons, Cyanobacteria, Protein engineering

## Abstract

**Background:**

Cyanobacteria produce hydrocarbons corresponding to diesel fuels by means of aldehyde-deformylating oxygenase (ADO). ADO catalyzes a difficult and unusual reaction in the conversion of aldehydes to hydrocarbons and has been widely used for biofuel production in metabolic engineering; however, its activity is low. A comparison of the amino acid sequences of highly active and less active ADOs will elucidate non-conserved residues that are essential for improving the hydrocarbon-producing activity of ADOs.

**Results:**

Here, we measured the activities of ADOs from 10 representative cyanobacterial strains by expressing each of them in *Escherichia coli* and quantifying the hydrocarbon yield and amount of soluble ADO. We demonstrated that the activity was highest for the ADO from *Synechococcus elongatus* PCC 7942 (7942ADO). In contrast, the ADO from *Gloeobacter violaceus* PCC 7421 (7421ADO) had low activity but yielded high amounts of soluble protein, resulting in a high production level of hydrocarbons. By introducing 37 single amino acid substitutions at the non-conserved residues of the less active ADO (7421ADO) to make its sequence more similar to that of the highly active ADO (7942ADO), we found 20 mutations that improved the activity of 7421ADO. In addition, 13 other mutations increased the amount of soluble ADO while maintaining more than 80% of wild-type activity. Correlation analysis showed a solubility-activity trade-off in ADO, in which activity was negatively correlated with solubility.

**Conclusions:**

We succeeded in identifying non-conserved residues that are essential for improving ADO activity. Our results may be useful for generating combinatorial mutants of ADO that have both higher activity and higher amounts of the soluble protein in vivo, thereby producing higher yields of biohydrocarbons.

**Electronic supplementary material:**

The online version of this article (10.1186/s13068-019-1409-8) contains supplementary material, which is available to authorized users.

## Background

The biosynthesis of hydrocarbons has provided a remarkable means of producing substitutes for petroleum-based fuels [1]. Biohydrocarbons are produced by various organisms, including bacteria, cyanobacteria, yeasts, plants, insects, birds, and green algae [2, 3]. Cyanobacteria-derived hydrocarbons have gained attention as a renewable energy source that can help limit global warming [4]. Moreover, the cyanobacterial biosynthesis of hydrocarbons is attractive because the growth rate of cyanobacteria is rapid, and their genetic manipulation is well established [5–10].

Cyanobacteria can convert fatty acyl–acyl carrier protein (ACP) and fatty acyl-coenzyme A (CoA), which are intermediates of fatty acid metabolism, into hydrocarbons that are 13–17 carbons in length, corresponding to diesel fuels, through two-step reactions [11]. The reactions are catalyzed by two enzymes, namely, acyl-ACP reductase (AAR) and aldehyde-deformylating oxygenase (ADO) [11, 12]. AAR reduces fatty acyl-ACPs or fatty acyl-CoAs to fatty aldehydes using NADPH [11, 13, 14]. The aldehyde products are delivered to ADO via the binding of AAR to ADO [14]. Subsequently, ADO converts saturated or monounsaturated fatty aldehydes (C_*n*_) into corresponding alkanes or alkenes (C_*n*−1_) and formate, respectively [11, 12]. *Escherichia coli* coexpressing cyanobacterial AAR and ADO can produce and secrete hydrocarbons [11]. Therefore, AAR and ADO are key enzymes for the biosynthesis of hydrocarbons. Thus, AAR and ADO have been applied for the production of biohydrocarbons in several organisms [10], including cyanobacteria [15–20], *E. coli* [11, 21–25], yeasts [26–28], chemoautotrophic eubacteria [29], and filamentous fungi [30]. In particular, ADO has been extensively used with other enzymes because ADO can catalyze a difficult and unusual reaction in the conversion of aldehydes to hydrocarbons [10, 23, 27, 31–35].

ADO is a member of a ferritin-like non-heme diiron family of enzymes, including methane monooxygenase, ribonucleotide reductase, and acyl-ACP desaturase [36]. ADO has eight α-helices, and its active site consists of two histidine and four carboxylate residues, with two iron atoms accommodated in an antiparallel four-helix bundle [37–40]. The substrate binding site is composed of a hydrophobic channel that terminates at the diiron center. An external reducing system composed of ferredoxin (Fd), ferredoxin NADP^+^ reductase (FNR), and NADPH is required for ADO activity [41]. Although ADO has been widely used for biofuel production by the metabolic engineering of cyanobacteria and other organisms, it has very low activity. Its maximum turnover number is ~ 1 min^−1^ [42], and the rate-limiting step in the biosynthesis of hydrocarbons in *E. coli* coexpressing AAR and ADO is the reaction catalyzed by ADO [10, 24]. There have been a few attempts to improve the hydrocarbon-producing activity of ADO by introducing amino acid substitutions based on the crystal structures of cyanobacterial ADOs [11, 22, 39]. These studies have revealed residues that are essential for maintaining the hydrocarbon-producing activity of ADO. Such residues include C83 located adjacent to the substrate binding site and those located near the diiron center [the residue numbering of ADO from *Prochlorococcus marinus* MIT 9313 (9313ADO) is used throughout this article]. In addition, several mutations at residues that compose the substrate binding channel have been reported to change the substrate specificity of ADO [33, 34, 43]. However, most of the residues at the mutation sites in these studies are highly conserved among ADOs from various cyanobacteria, and there have been few reports on mutations at non-conserved residues. Because the amino acid residues of an enzyme are generally conserved to maintain its activity [44–46] and because functionally important residues are generally less mutationally tolerant than residues with less stringent functional constraints [46–48], mutations that improve enzymatic activity are expected to be introduced mainly at non-conserved residues. Thus, to improve the hydrocarbon-producing activity of ADO, a survey of amino acid substitutions at non-conserved sites is necessary. A comparison of the amino acid sequences of highly active and less active ADOs will help to elucidate the mutations at non-conserved residues that are essential for improving hydrocarbon-producing activity.

To compare the activity of the ADO mutants, a method that can accurately measure ADO activity must be employed. However, accurate measurements of ADO activity in vitro have not been possible because the natural substrates of ADO, namely, medium- and long-chain (C_10_–C_18_) aldehydes, have low solubility and form micelles in solution [14]. Consequently, the dissociation reaction of an aldehyde molecule from micelles is the rate-determining step in the overall reaction and obscures the catalytic reaction of ADO [43, 49–51]. Instead, ADO activity for medium- and long-chain aldehydes has been measured in vivo by quantifying the hydrocarbon production yield [11, 16, 22, 52–54]. Several studies have shown that ADO from *Nostoc punctiforme* PCC 73102 (73102ADO) or *Synechococcus elongatus* PCC 7942 (7942ADO) produces the highest yield of hydrocarbons when ADO is expressed in *E. coli*, yeast, or cyanobacteria [11, 28, 35, 53]. In contrast, other studies have reported that ADOs from *Thermosynechococcus elongatus* BP-1 (*Te*ADO), *Cyanothece* sp. PCC 7425 (7425ADO), and *Planktothrix agardhii* NIVA-CYA 126/8 (*Pa*ADO) produce hydrocarbons in amounts that are higher than or comparable to those produced by 73102ADO and 7942ADO [28, 52, 54], which contradicts previous reports. The inconsistency of these results may be attributed to the methods used for evaluating ADO activity. All of these studies compared the yield of hydrocarbons without considering the amount of the soluble form of ADO inside the cells. However, because the hydrocarbon production yield is directly related to both ADO activity and the amount of the soluble form of ADO, it is necessary to measure the amount of soluble ADO to accurately evaluate ADO activity.

Previously, we established a method for accurate measurements of ADO activity [22]. In this method, both AAR and ADO are coexpressed in *E. coli*, and fatty aldehyde substrates are supplied from AAR to ADO. ADO activity was calculated as the amount of hydrocarbon produced [quantified using gas chromatography–mass spectrometry (GC–MS)] divided by the amount of the soluble form of ADO [quantified using sodium dodecyl sulfate-polyacrylamide gel electrophoresis (SDS-PAGE) or western blotting]. The same method has been successfully applied to measure the activity of AAR, and we found that AAR from *Synechococcus elongatus* PCC 7942 (7942AAR) had the highest activity among the examined AARs [24]. Thus, our method can be applied to accurately determine the activity of various ADOs and to identify residues that are essential for increasing ADO activity. In this method, factors affecting the hydrocarbon-producing activity of ADO include the catalytic efficiency of ADO, the efficiency of substrate delivery from AAR to ADO by their interactions, and the efficiency of coupling between ADO and the Fd/FNR/NADPH reducing system. Thus, this method can identify ADO residues that are essential for improving hydrocarbon-producing activity in terms of these factors.

Here, using this method, we compared the hydrocarbon-producing activity of ADOs from 10 representative cyanobacterial strains. 7942AAR, which has the highest activity among the various AARs [24], was used as a supplier of aldehyde substrates. We found that 7942ADO has the highest activity. In contrast, ADO from *Gloeobacter violaceus* PCC 7421 (7421ADO) has low activity but yields high amounts of the soluble protein, resulting in a high production level of hydrocarbons. By constructing 37 mutants of 7421ADO, in which non-conserved residues were substituted into the amino acids used in 7942ADO, we identified non-conserved residues that are responsible for the high activity of ADO, as well as mutations that increase the amount of soluble ADO in *E. coli*. Our results may be useful for generating combinatorial mutants of ADO that have both higher activity and higher amounts of the soluble protein in vivo, thereby producing a higher yield of biohydrocarbons.

## Results

### Selection of 10 representative ADOs

**Fig. 1:**
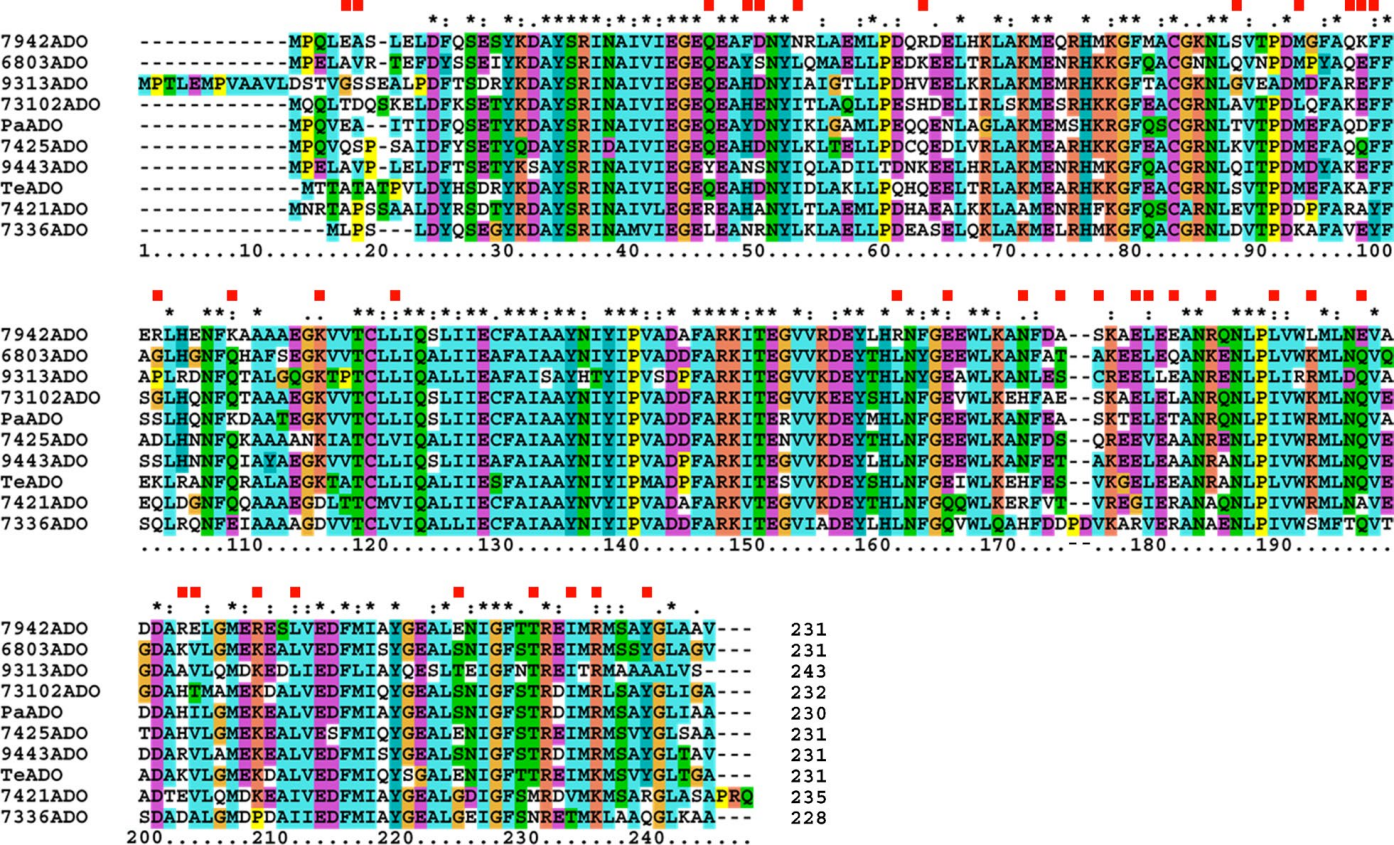
Amino acid sequences of 10 representative ADOs. The asterisk denotes completely conserved residues, and the colon and dot denote partially conserved residues. Red squares indicate the mutation sites of the 7421ADO mutants used in the present study. The amino acid sequences of ADOs are shown in descending order of activity. The residue numbering of ADO from 9313ADO is shown at the bottom of the sequence alignments

Previously, we compared the aldehyde-producing activity of AARs from eight representative cyanobacteria [24]. Here, we compared the activity of the ADOs from the same eight representative cyanobacteria: 73102ADO, 7421ADO, 7942ADO, 9313ADO, and *Te*ADO and ADOs from *Synechocystis* sp. PCC 6803 (6803ADO), *Synechococcus* sp. PCC 7336 (7336ADO), and *Microcystis aeruginosa* PCC 9443 (9443ADO) [24]. In addition, we also used 7425ADO and *Pa*ADO because these ADOs have been reported to produce high amounts of hydrocarbons [28, 52]. Thus, a total of 10 ADOs were selected for the activity assays. Multiple sequence alignments of the 10 ADOs are shown in Fig. 1. The amino acid sequence identities among them are, on average, 69% (Additional file 1: Table S1).

A phylogenetic tree of cyanobacterial ADOs was constructed based on the ADO amino acid sequences (Additional file 2: Figure S1). There are two large groups (groups 1 and 2) and one small group (group 3) of ADO sequences in the phylogenetic tree, which is similar to that constructed using AAR amino acid sequences [24]. Group 1 contains 93 ADO sequences, which are mainly from freshwater cyanobacteria, including *Nostoc punctiforme* PCC 73102, *Synechococcus elongatus* PCC 7942, *Planktothrix agardhii* NIVA-CYA 126/8, *Synechocystis* sp. PCC 6803, *Microcystis aeruginosa* PCC 9443, *Cyanothece* sp. PCC 7425, and *Thermosynechococcus elongatus* BP-1. Group 2 contains 31 ADO sequences, which are mainly from marine cyanobacteria, including *Prochlorococcus marinus* MIT 9313. Group 3 contains 10 ADO sequences from both marine and freshwater cyanobacteria, such as *Synechococcus* sp. PCC 7336 and *Gloeobacter violaceus* PCC 7421, respectively. The amino acid sequences of the ADOs from Group 3 are distinct from those categorized into groups 1 and 2 (Additional file 1: Table S1).

### ADO activity

ADO activity towards the C_16_ and C_18_ aldehydes was measured with an in vivo activity assay that we previously developed [22, 24] because the in vitro activity measurement was precluded by the low solubility of the substrates (see above). Thus, we coexpressed 7942AAR, having the highest activity among the various AARs [24], with one of the 10 selected ADOs in *E. coli* to produce hydrocarbons. In this method, ADOs that exhibit higher hydrocarbon-producing activity should have higher catalytic efficiency, higher affinity for 7942AAR, and/or more efficient coupling with an external reducing system. A GC–MS profile of the extract from the *E. coli* cell culture coexpressing AAR and ADO showed predominant amounts of heptadecene and pentadecane and a small amount of heptadecane (Additional file 3: Figure S2), consistent with previous studies [22, 24]. The predominant production of heptadecene (alkene) is probably related to the fact that most acyl-ACPs are desaturated during fatty acid synthesis in *E. coli* [55].

A control experiment showed that fatty aldehydes and corresponding fatty alcohols can be extracted using ethyl acetate (Additional file 4: Figure S3a, b). Thus, the GC–MS profiles of the extracts from the *E. coli* cell cultures coexpressing AAR and ADO showed peaks of fatty aldehydes and fatty alcohols, in addition to those of alkanes/alkenes (Additional file 4: Figure S3d). However, because the peaks for aldehydes and alcohols had extremely low intensities and were partially overlapped with those from *E. coli* cells (Additional file 4: Figure S3c, d), it was not possible to accurately quantify their amounts and therefore to estimate ADO activity using them.

**Fig. 2:**
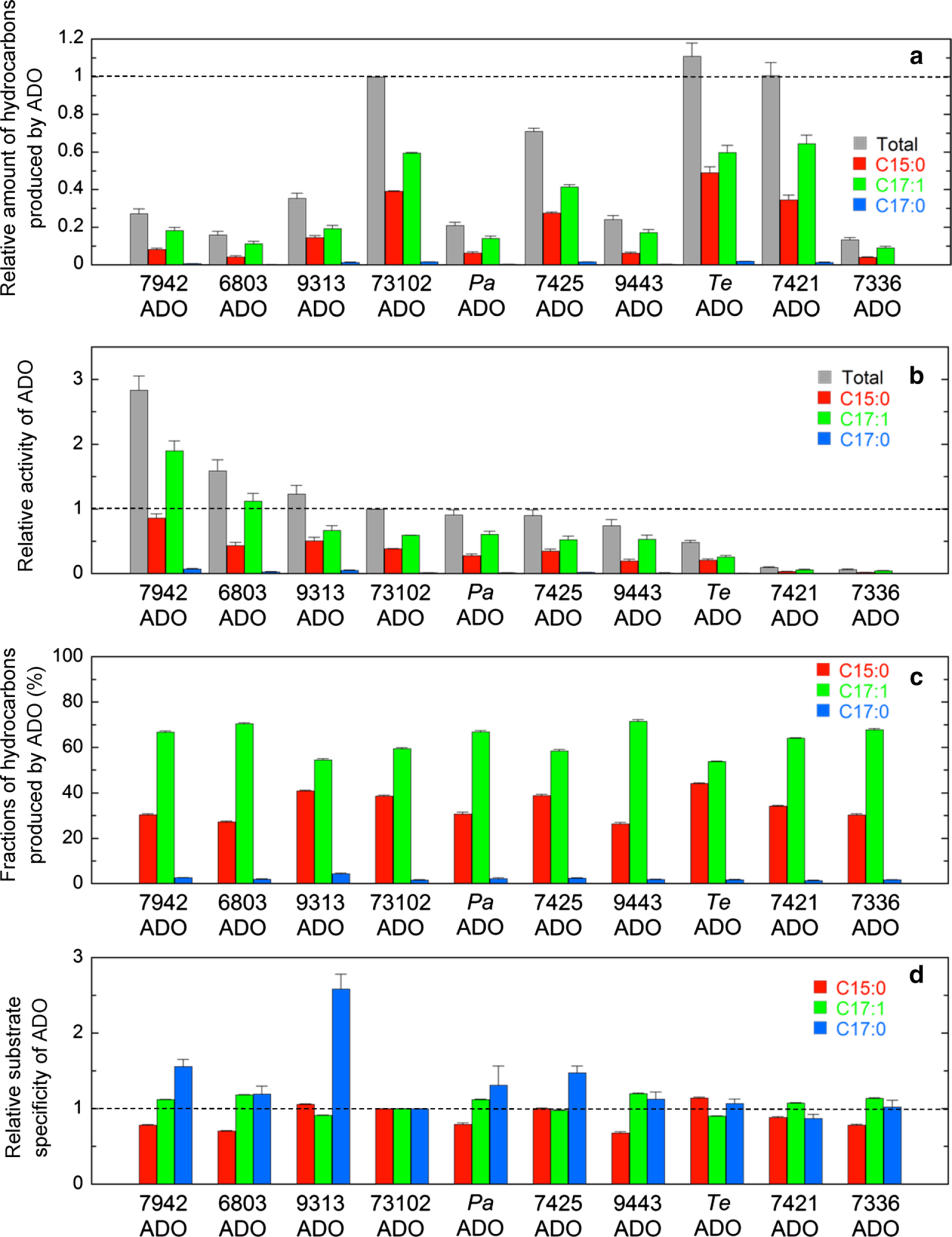
Activity and substrate specificity of 10 representative ADOs. **a** The amounts of hydrocarbons produced in *E. coli* coexpressing ADO and 7942AAR. The amounts of pentadecane (C15:0), heptadecene (C17:1), and heptadecane (C17:0) and their combined total amount are shown in red, green, blue, and gray, respectively. The values relative to the total amount of hydrocarbon produced in *E. coli* coexpressing 73102ADO and 7942AAR are shown. **b** The hydrocarbon-producing activity of ADO relative to that of 73102ADO. The values are expressed as the amount of hydrocarbon divided by the amount of the soluble form of ADO. **c** Fractions of pentadecane, heptadecene, and heptadecane relative to the total amount of hydrocarbon produced in *E. coli*. **d** The substrate specificity of ADO relative to that of 73102ADO. In all panels, the data are shown in descending order of activity. A horizontal dotted line shows the value for 73102ADO. The measurements were carried out more than three times, and the means ± standard errors are shown

Figure 2a shows the amounts of pentadecane, heptadecene, and heptadecane and their total amounts in *E. coli* cell cultures coexpressing ADO from one of 10 representative cyanobacteria and 7942AAR. Because 73102ADO had been previously considered to have the highest activity [11], the amounts of hydrocarbons relative to those produced by 73102ADO are shown in Fig. 2. The actual amounts of produced hydrocarbons are shown in Additional file 5: Table S2. The total amounts of hydrocarbons produced in *E. coli* were the highest for *Te*ADO, followed by, in descending order, 7421ADO, 73102ADO, 7425ADO, 9313ADO, 7942ADO, 9443ADO, *Pa*ADO, 6803ADO, and 7336ADO. The amounts of hydrocarbons produced by 6803ADO and 7336ADO were less than 20% of that produced by *Te*ADO.

**Fig. 3:**
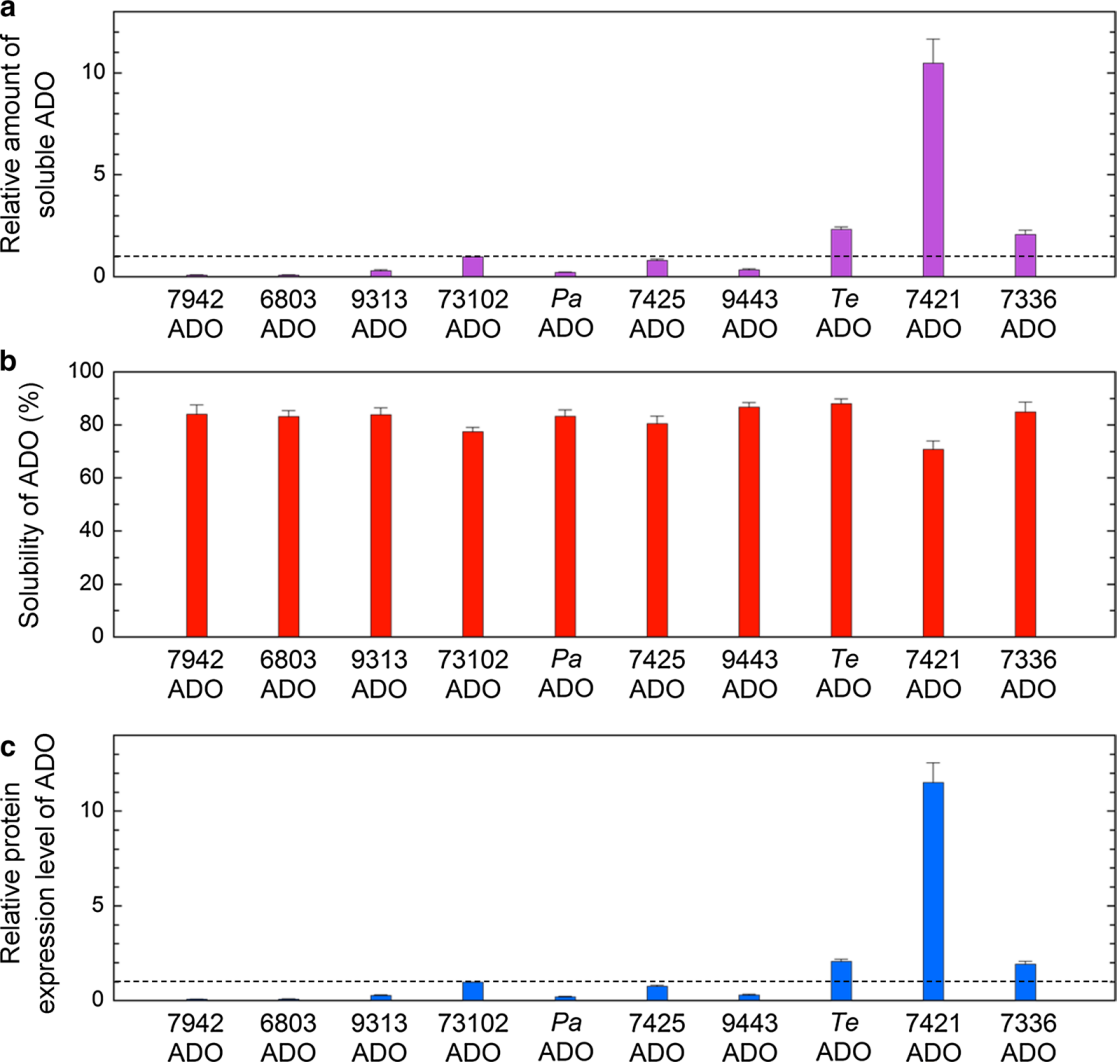
Solubility and protein expression levels of 10 representative ADOs quantified by western blotting. **a** The amount of the soluble form of ADO normalized to that of 73102ADO. **b** The solubility (%) of ADO calculated as the ratio of the amount of the soluble form to the total amount of the soluble and insoluble forms of ADO. **c** The protein expression level of ADO, calculated as the total amount of the soluble and insoluble forms, is normalized to that of 73102ADO. In all panels, the data are shown in descending order of activity. A horizontal dotted line shows the value for 73102ADO. The measurements were carried out more than three times, and the means ± standard errors are shown

**Fig. 4:**
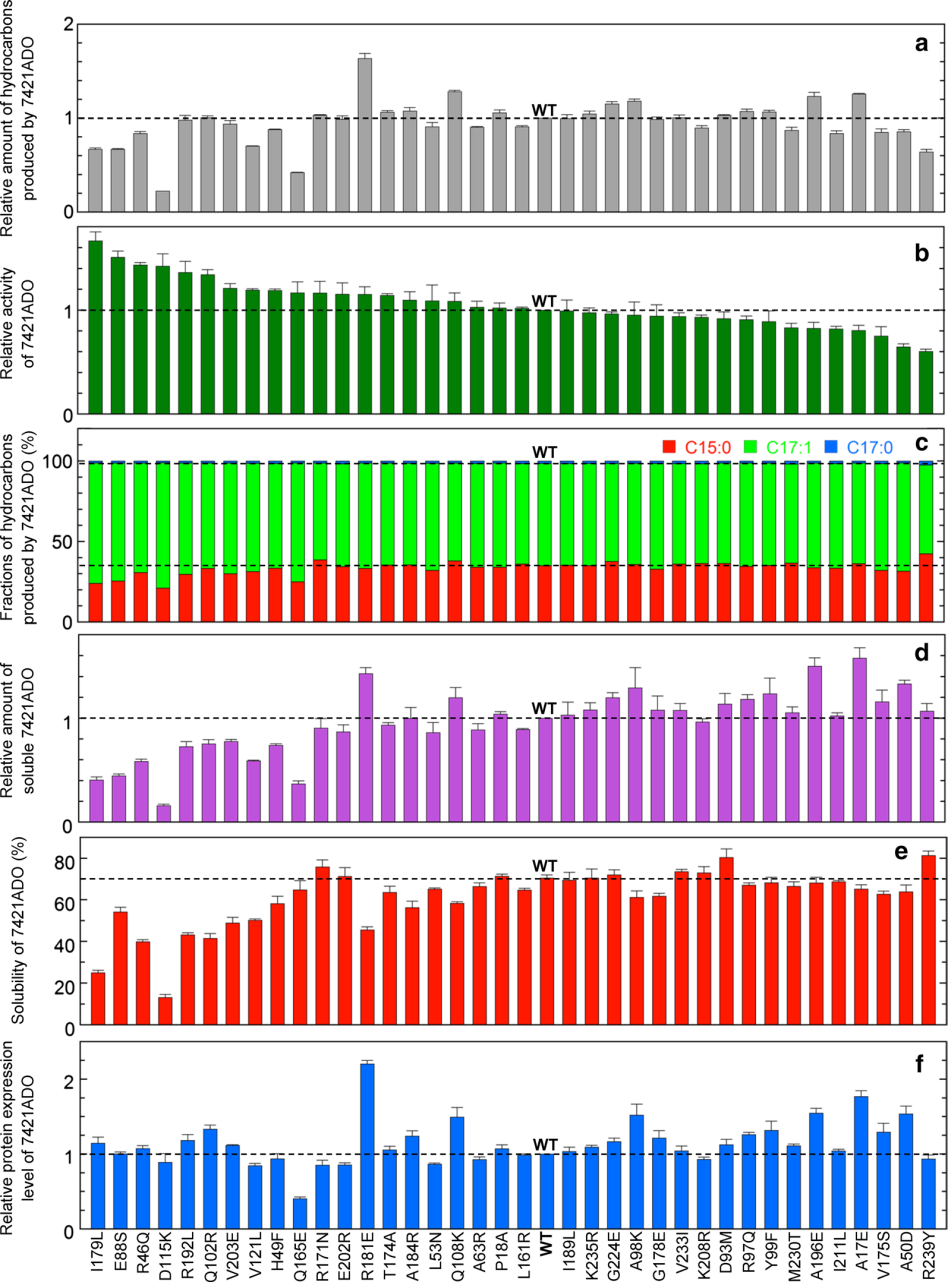
Activity and solubility of mutants of 7421ADO. **a** The amount of total hydrocarbon produced in *E. coli* coexpressing 7942AAR and a mutant of 7421ADO relative to that of wild-type (WT) 7421ADO. **b** The hydrocarbon-producing activity of a 7421ADO mutant relative to that of the WT. **c** Fractions of pentadecane, heptadecene, and heptadecane relative to the total amount of hydrocarbon produced by a 7421ADO mutant. **d** The amount of the soluble form of a 7421ADO mutant relative to that of the WT. **e** Solubility (%) of ADO. **f** The protein expression level of 7421ADO relative to that of the WT. In all panels, the data are shown in descending order of activity. A horizontal dotted line shows the value for the WT. The measurements were carried out more than three times, and the means ± standard errors are shown

Factors affecting the amounts of hydrocarbons produced in *E. coli* are ADO activity and the amount of the soluble form of ADO. Therefore, the total amount of hydrocarbon in the *E. coli* cell culture, normalized to the amount of the soluble form of ADO, was used as an index of ADO activity. The amount of the soluble form of ADO was quantified by western blotting (Fig. 3a; Additional file 6: Figure S4) because western blotting and SDS-PAGE have been successfully used to quantify the amounts of soluble and insoluble forms of proteins [56–58]. The 7425ADO protein migrated more slowly than the other ADOs. This may be because the 7425ADO protein has the highest number of negative charges at pH 8.8 among the ADOs used in this study (see Additional file 7: Table S3), as abnormally slow migration by SDS-PAGE has been previously reported for proteins that have many negative charges [59, 60]. The western blotting results showed that there were large differences in the amount of the soluble form among the various ADOs. We found that the amount of the soluble protein was high for 7421ADO, *Te*ADO, and 7336ADO but low for *Pa*ADO, 6803ADO, and 7942ADO (Fig. 3a; Additional file 6: Figure S4). Notably, the variations in the amounts of soluble ADOs were not due to handling errors associated with western blotting because the reproducibility of the data was confirmed by triplicate experiments, as shown in Additional file 6: Figure S4a; the error bars for quantifications of the amounts of soluble ADO and AAR are shown in Fig. 4 and Additional file 6: Figure S4b, respectively.

Figure 2b shows the hydrocarbon-producing activity of ADO expressed as the total amount of hydrocarbons in the *E. coli* cell culture (Fig. 2a) divided by the amount of the soluble form of ADO (Fig. 3a). The activities relative to that of 73102ADO are shown. The hydrocarbon-producing activity was the highest for 7942ADO, followed by, in descending order, 6803ADO, 9313ADO, 73102ADO, *Pa*ADO, 7425ADO, 9443ADO, *Te*ADO, 7421ADO, and 7336ADO (Fig. 2b). Although the amount of the soluble form of ADO for 7421ADO, *Te*ADO, and 7336ADO was high, their activities were low.

Additional file 6: Figure S4b shows that the amount of soluble AAR ranged from 80 to 175% when the value of *E. coli* coexpressing 7942AAR and 73102ADO was used as a reference. However, this variation does not affect the measurement of ADO activities. We have previously shown that the rate-limiting step in the biosynthesis of hydrocarbons in *E. coli* coexpressing 7942AAR and 73102ADO is the reaction catalyzed by ADO [24]. Moreover, when the amount of the soluble AAR protein was reduced to 1/3 (33%), the amount of hydrocarbon produced by 73102ADO was decreased to 1/2 [24]. This result indicates that when the amount of soluble AAR is reduced to 2/3 (66%), the efficiency of substrate supply by AAR becomes the same as that of hydrocarbon production by 73102ADO. Thus, if the amount of soluble AAR is larger than 80%, aldehyde substrates that are sufficient for a hydrocarbon production level that is 1.2-fold higher than that of 73102ADO are supplied. Figure 2a shows that the levels of hydrocarbons produced by all ADOs were less than 1.2-fold that of 73102ADO. Therefore, in the present study, the aldehyde supply from AAR was sufficient for estimating ADO activities, and normalizing the amount of hydrocarbon to the amount of soluble AAR was unnecessary when estimating ADO activity.

### Solubility and protein expression level

The solubility and protein expression levels of the ADOs in *E. coli* were quantified by western blotting (Fig. 3b, c). Here, the protein expression level was determined as the total amount of the soluble and insoluble forms of ADO, while solubility was estimated as the ratio of the amount of the soluble form to the total amount of the soluble and insoluble forms of ADO. We found that solubility was highest for *Te*ADO (88%), followed by, in descending order, 9443ADO, 7336ADO, 7942ADO, 9313ADO, *Pa*ADO, 6803ADO, 7425ADO, 73102ADO, and 7421ADO (Fig. 3b). The high solubility of *Te*ADO may be explained by the fact that proteins derived from thermophilic bacteria have high stability [61]. In contrast, the solubility of 7421ADO was lowest (70%). This may be due to the significantly high expression level of the 7421ADO protein (Fig. 3c). Nonetheless, the amount of the soluble form of 7421ADO, which corresponds to the product of the protein expression level and solubility, was highest among the ADOs (Fig. 3a).

### Substrate specificity

All 10 ADOs examined in this study showed that their major product is heptadecene (Fig. 2c), indicating that these ADOs have a similar substrate specificity. However, there were slight differences in the substrate specificities of the 10 ADOs (Fig. 2d). 9443ADO and 6803ADO had relatively higher substrate specificities for the 18-carbon fatty aldehyde than the other ADOs because the amount of pentadecane was less than 30% of the total amount of produced hydrocarbon (Fig. 2c). In contrast, the amount of pentadecane was more than 40% of the total amount of hydrocarbon produced with *Te*ADO and 9313ADO, which are derived from freshwater and marine cyanobacteria, respectively (Fig. 2c), indicating that these ADOs had a relatively higher substrate specificity for the 16-carbon fatty aldehyde. Among the three types of detected hydrocarbons, heptadecane exhibited the lowest production (Fig. 2a). Nevertheless, the level of heptadecane produced by 9313ADO was ~ 2.5 times higher than that produced by 73102ADO (Fig. 2d).

### Correlation analysis

Correlation analysis of activity, solubility, the protein expression level, the amount of hydrocarbon, and the amount of soluble ADO measured for the 10 representative ADOs was performed (Additional file 8: Figure S5). Strong correlations were not observed, except for a correlation between the protein expression level and the amount of soluble ADO. However, clear correlations between ADO properties were observed when the data from the 7421ADO mutants were used (see below).

We also carried out correlation analyses between the above properties and the amino acid sequence identities. To determine whether the activity was higher for the ADOs with amino acid sequences that were more similar to that of the most active ADO, we plotted the ADO activities against the sequence identities with 7942ADO, which exhibited the highest activity (Additional file 9: Figure S6a). There appears to be a positive correlation, but when the data point for 7942ADO is omitted, the correlation disappears, indicating that the activity level of ADO is not determined by the overall similarity of amino acid sequences. Similarly, solubility, the relative protein expression level, the relative amount of hydrocarbon, and the relative amount of soluble ADO do not correlate well with the sequence identities of *Te*ADO, 7421ADO, *Te*ADO, and 7421ADO, respectively, which have the highest number of corresponding properties (Additional file 9: Figure S6b–e). These results indicate that local differences in amino acid sequences, which cannot be inferred from the phylogenetic tree of ADOs, determine these ADO properties, suggesting that a limited number of non-conserved residues determine the activity level of an ADO.

### Mutational analysis of non-conserved residues of 7421ADO

As shown above, hydrocarbon-producing activity is diverse among the various ADOs. This difference can be ascribed to the residues not being completely conserved among ADOs. To identify the non-conserved residues that are essential for ADO activity, we introduced mutations at the non-conserved residues of the less active ADO (7421ADO) to make its amino acid sequence more similar to that of the highly active ADO (7942ADO). The sites for mutation were selected using the multiple sequence alignment shown in Fig. 1, in which the amino acid sequences of the ADOs are shown in descending order of activity. Eighty-four ADO residues are completely conserved. Among the non-conserved residues, including partially conserved residues, both 7421ADO and 7942ADO have the same amino acids at 56 positions, which were excluded from the group of candidates for mutational studies. In addition, positions excluded from the group of candidates were those with the same amino acids as the highly active 7942ADO or the less active 7336ADO. Thus, we selected 37 positions in 7421ADO and replaced them with the corresponding residues in 7942ADO one at a time (Fig. 1). We then coexpressed each of the 37 mutants of 7421ADO with 7942AAR in *E. coli* and measured the amounts of produced hydrocarbons and soluble ADO, as well as the activity, substrate specificity, solubility, and protein expression level of the ADO (Fig. 4).

As expected, mutations affected the hydrocarbon-producing activity of 7421ADO. Twenty out of 37 mutations improved ADO activity (Fig. 4b). Among them, four mutations increased the activity of all hydrocarbons by more than 40% (I179L, E88S, R46Q, and D115K mutations, in descending order of activity). Six other mutations increased the activity by more than 17% (R192L, Q102R, V203E, V121L, H49F, and Q165E). However, the amount of the soluble form of ADO was decreased in the 10 mutants (Fig. 4d). These results are consistent with the fact that 7942ADO has high activity but yields low amounts of the soluble protein (Figs. 2, 3). Ten other mutations increased the activity by up to 17% but maintained more than 85% of the amount of soluble ADO (R171N, E202R, R181E, T174A, A184R, L53N, Q108K, A63R, P18A, and L161R) (Fig. 4b, d).

In contrast, 17 mutations in 7421ADO decreased the activity while increasing or maintaining the amount of soluble ADO (Fig. 4b, d). Among them, 13 mutations had more than 80% wild-type activity while increasing the amount of soluble protein (I189L, K235R, G224E, A98K, G178E, V233I, D93M, R97Q, Y99F, M230T, A196E, I211L, and A17E). However, three mutations (R239Y, A50D, and V175S) decreased the activity by more than 20%.

Substrate specificity was almost unchanged by the mutations, although the fraction of pentadecane was slightly decreased for the mutants having the 10 highest activities (Fig. 4c). The results are consistent with the fact that all representative ADOs have similar specificity for the substrates (Fig. 2c).

In addition to their effects on activity, the mutations also affected the amounts of hydrocarbons produced by ADO. Among the 37 mutants examined, the R181E mutant showed the highest yield of total hydrocarbon, which was 60% higher than that of the wild-type 7421ADO (Fig. 4a). Subsequently, a more than 20% increase in hydrocarbon production was observed for the Q108K, A17E, A196E, A98K, and G224E mutants of 7421ADO, in descending order. However, the activities of these mutants, especially those of A17E and A196E, both of which had activities of ~ 0.8-fold that of the wild type (Fig. 4b), did not increase by much, and the amount of the soluble form of ADO was increased by more than 20% (Fig. 4d). In particular, A17E showed the highest amount of soluble ADO among all mutants (57% increase compared with that of the wild type), followed by A196E and R181E.

The amount of the soluble form of ADO depends on both the solubility and protein expression level of ADO. A decrease in solubility was observed for the mutants with high activities (Fig. 4e). For some mutants, the protein expression level was increased by more than 50%, especially for R181E (2.2-fold greater than that of the wild type) (Fig. 4f). This increase in the protein expression level resulted in the higher amount of soluble ADO (Fig. 4d).

### Correlation analysis among the properties of 7421ADO mutants

**Fig. 5:**
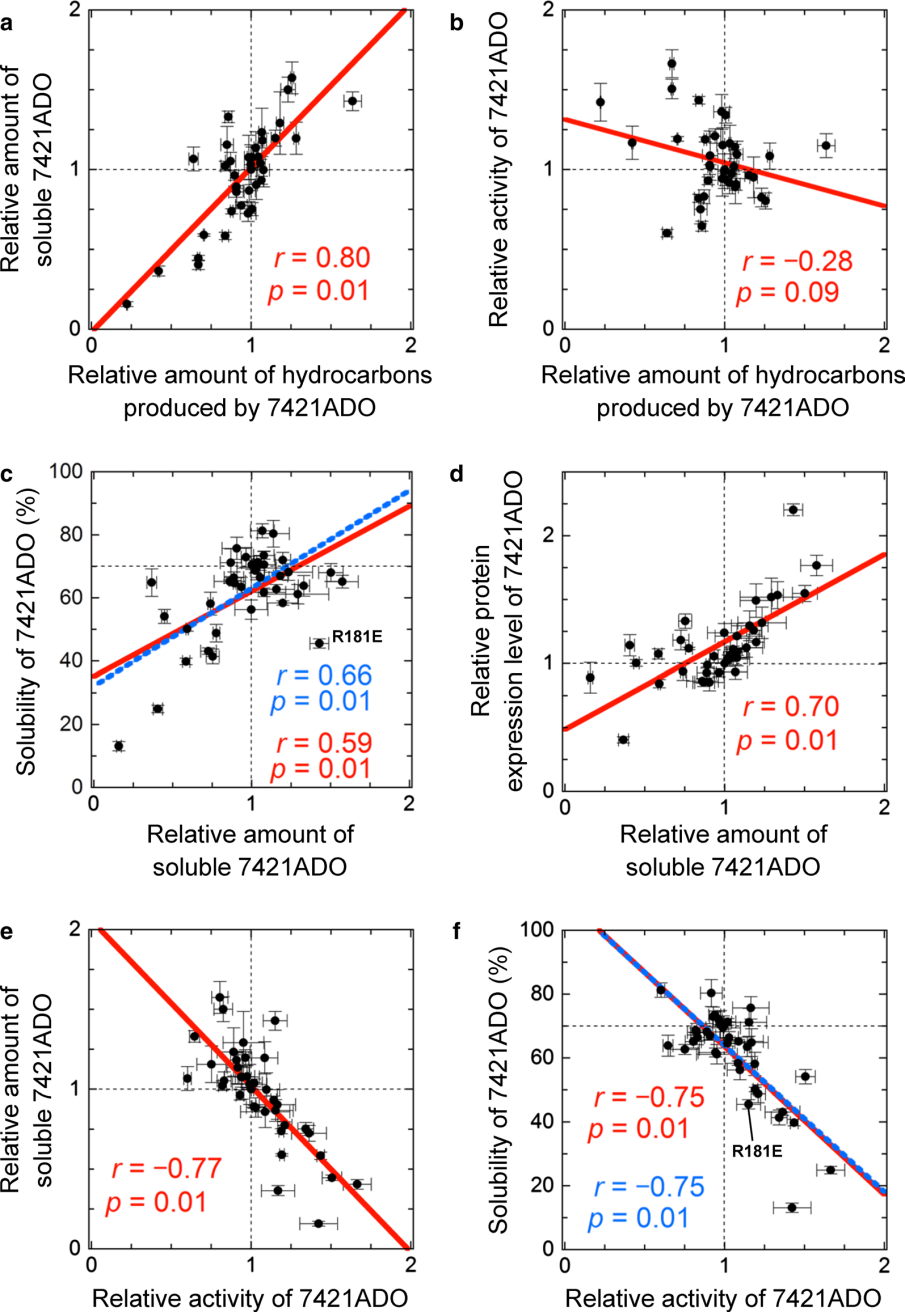
Correlation analysis of the mutants of 7421ADO. **a**, **b** The relative amount of total hydrocarbon produced in *E. coli* coexpressing 7942AAR and a mutant of 7421ADO plotted against the relative amount of soluble ADO (**a**) and relative activity for total hydrocarbons (**b**). **c**, **d** The relative amount of soluble 7421ADO plotted against the solubility (**c**) and relative protein expression level of 7421ADO (**d**). **e**, **f** The relative activity for total hydrocarbons of 7421ADO plotted against the relative amount of soluble 7421ADO (**e**) and solubility (**f**). In each panel, the red continuous line indicates a linear regression, and the corresponding correlation coefficient, *r*, and *p* value are shown. In **c**, **f**, the data point for R181E is highlighted. The blue dotted line indicates a linear regression obtained without using the data for R181E

We performed a correlation analysis among the properties measured for the 37 mutants of 7421ADO (Fig. 5; Additional file 10: Figure S7). The amounts of total hydrocarbons produced in *E. coli* cells coexpressing AAR and ADO should be directly related to both the activity of the ADO and the amount of the soluble form of ADO. We found that there was a good positive correlation between the amount of total hydrocarbon and the amount of the soluble form of ADO (Fig. 5a). In contrast, a clear correlation was not observed between the amount of hydrocarbon and activity (Fig. 5b).

The amount of the soluble form of ADO should be directly related to both the solubility and protein expression level of the ADO. In fact, the amount of the soluble form was positively correlated with both the solubility and protein expression level of the ADO (Fig. 5c, d). In contrast, the activity of the ADO was negatively correlated with both the amount of soluble ADO and solubility (Fig. 5e, f) but not with the protein expression level of the ADO (Additional file 10: Figure S7c).

The correlation between the amount of the soluble form and solubility (Fig. 5c) may seem to contradict the results for the R181E mutant, which showed higher amounts of the soluble protein but had lower solubility than the wild-type 7421ADO. However, this may be due to the twofold higher expression level of the protein relative to wild-type 7421ADO expression, and the solubility of R181E may not be correctly estimated. When this data point is omitted from Fig. 5c, a more significant correlation is observed between solubility and the amount of soluble ADO. Similarly, the correlation between the solubility and the amount of hydrocarbon produced by the ADO becomes more significant when the data point for R181E is omitted (Additional file 10: Figure S7a).

## Discussion

### Comparison of various ADOs

Comparing the activities of ADOs from various cyanobacteria is important when selecting an ADO that is suitable for hydrocarbon production. However, there have been no studies in which an accurate comparison of ADO activity has been performed by normalizing hydrocarbon production to the amount of the soluble form of ADO. Here, we measured both the hydrocarbon production yield and the amount of the soluble form of ADO and accurately evaluated ADO activity. We demonstrated that the hydrocarbon-producing activity is highest for 7942ADO (Fig. 2). However, since the amount of the soluble protein was low for 7942ADO, the total amount of hydrocarbon produced in *E. coli* expressing 7942ADO together with 7942AAR was moderate among the 10 ADOs examined.

In contrast, 7421ADO, *Te*ADO, and 73102ADO had low activity but showed both high amounts of the soluble ADO protein and high yields of hydrocarbon production. In particular, the high amount of the soluble protein for 7421ADO compensated for its low activity and resulted in high amounts of produced hydrocarbons. Similarly, *Te*ADO yielded the second highest amount of soluble protein (Fig. 3a) and the highest amount of hydrocarbon (Fig. 2a). In general, enzymes from thermophilic bacteria have high optimal temperatures and low activity at 25–37 °C due to the slower dynamics of the enzymes [62]. Thus, *Te*ADO may be useful when hydrocarbon production is conducted at higher temperatures. Finally, consistent with previous reports [11, 50, 53], 73102ADO produced a high amount of hydrocarbon. However, our results also indicate that this was not due to the high activity of the enzyme but to both a moderate activity and a moderate expression level of the 73102ADO protein.

Aldehydes produced by AAR have been reported to be efficiently delivered to ADO by the binding of AAR to ADO [14]. It is possible that AAR and ADO that are derived from the same strain interact with each other more efficiently than those derived from different strains, resulting in a higher production of hydrocarbons. In accordance with this, the combination of AAR and ADO, both of which are derived from *Synechococcus elongatus* PCC 7942, showed the highest activity (Fig. 2b). However, when 73102ADO was coexpressed with one of 12 representative AARs, the highest AAR activity was observed for 7942AAR, but not for AAR from *Nostoc punctiforme* PCC 73102 [24]. These results suggest that AAR and ADO derived from the same strain do not always interact more tightly than those derived from different strains.

Unexpectedly, we observed a variation in the amount of soluble 7942AAR protein depending on the coexpressed ADO (Additional file 6: Figure S4). It may be possible that the interaction between AAR and ADO stabilizes the AAR protein, resulting in an increase in the amount of soluble AAR. If this is the case, the present results might suggest that 9313ADO, with which the highest amount of soluble 7942AAR was coexpressed, binds 7942AAR more tightly than other ADOs and stabilizes it, leading to high accumulation of AAR.

### Mutational analysis of ADO

In the present mutational analysis of 7421ADO, we identified 20 non-conserved residues that increased ADO activity. However, the mutants that increased the activity showed a decrease in hydrocarbon production due to reductions in their solubility and, therefore, the amount of soluble ADO. There was a good negative correlation between activity and solubility (Fig. 5f). It has been reported that there is a trade-off between the stability and activity of an enzyme [63]. Because a protein exerts its function through dynamic motions, an enzyme with high activity shows enhanced structural flexibility [64], which results in a decrease in stability, leading to aggregation and lower solubility.

We also identified mutations that increased hydrocarbon production by introducing single substitutions at non-conserved residues (i.e., R181E, Q108K, A17E, A196E, A98K, and G224E). The highest and second highest hydrocarbon production levels were observed for the R181E and Q108K mutants, which increased both the activity by 15% and 9% and the amount of soluble protein by 43% and 20%, respectively, compared with those of the wild type. Other mutants that produced 10% or more hydrocarbon than the wild type had more soluble ADO than and similar activity to the wild type. These results suggest that mutations that increase both the activity and the amount of the soluble protein are the most effective in increasing hydrocarbon production and that the mutations that increase either the activity or the amount of the soluble protein while maintaining the amount of the soluble protein or activity, respectively, are the second most effective.

In addition to R181E and Q108K, there were eight mutations that improved ADO activity while maintaining more than 85% of the amount of soluble ADO (Fig. 4). Moreover, 13 other mutations increased the amount of soluble ADO while maintaining more than 80% of wild-type activity. In particular, A17E and A196E increased the amount of soluble ADO by more than 50% compared with that of the wild type. A combination of these mutations may enable us to generate 7421ADO mutants that have higher activity and yield higher amounts of the soluble protein in vivo, leading to higher production of hydrocarbons.

In addition to the mutations at non-conserved residues, appropriate mutations at conserved residues might improve the activity and solubility of ADO. Previous studies have shown that although mutations at conserved residues decrease enzymatic activity in many cases [46–48], conserved residues located far from the active site of a given protein may provide targets for substitution to improve protein properties [39, 65].

### Mapping the mutation sites onto the ADO structure

**Fig. 6:**
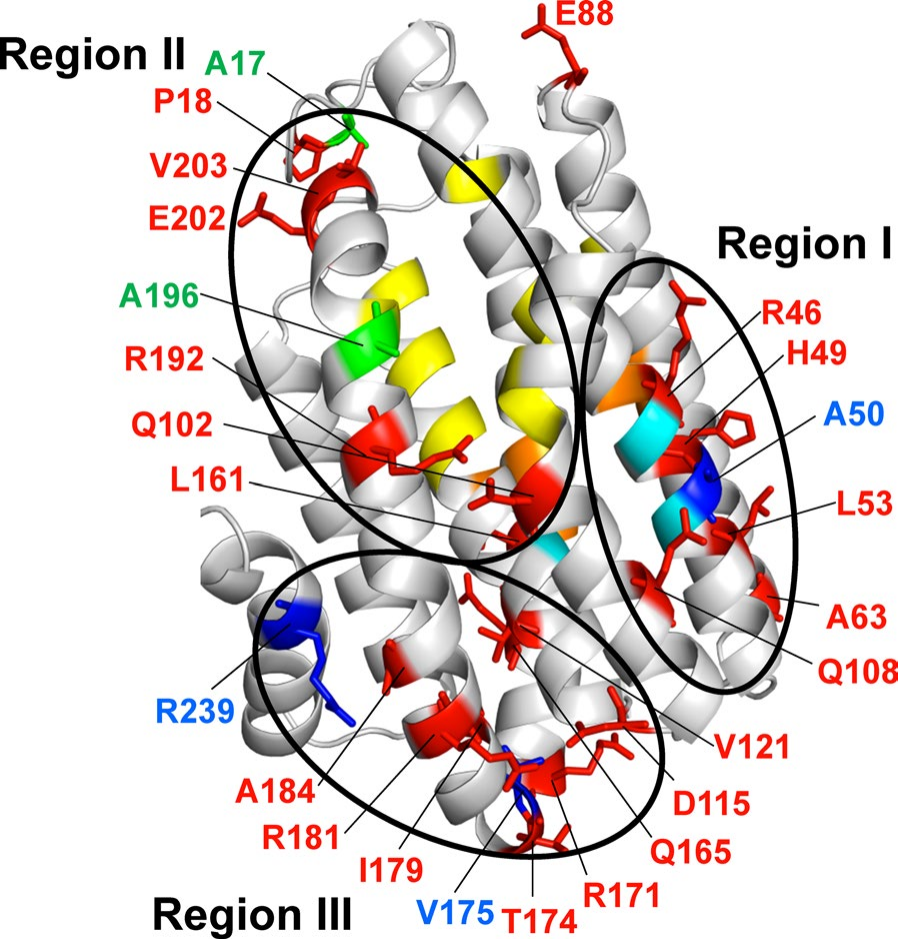
Map of the mutation sites onto the structure of 7421ADO. The structure was built by homology modeling using I-TASSER [66]. The substrate binding site (yellow), iron binding sites (orange), and proton supply channel to the active center (cyan) are shown in the structure [10]. The mutation sites of 20 mutants that increased activity, 3 mutants that decreased activity by more than 20%, and 2 mutants that increased the amount of soluble ADO by more than 50% are indicated by red, blue, and green sticks, respectively. The three regions are indicated by black circles

To understand the effects of mutations on ADO from the viewpoint of the three-dimensional structure, we mapped the mutation sites onto the structure of 7421ADO. Although X-ray crystal structures have been determined for 9313ADO [37, 43], 7942ADO [38], and 6803ADO [39], the structure of 7421ADO has not been solved. Thus, the structure of 7421ADO was constructed by homology modeling using the I-TASSER server [66]. The mutation sites of 20 mutants that increased activity, 3 mutants that decreased activity by more than 20%, and 2 mutants that increased the amount of soluble ADO by more than 50% compared with the wild type were mapped onto the ADO structure (Fig. 6). All of these mutation sites, except for E88, can be categorized into the following three regions.

Region I, including R46, H49, A50, L53, A63, and Q108, is close to the residues involved in the putative proton relay pathway (composed of E47, N51, E108, and E123) from the solvent to the diiron center [37] (Fig. 6). In particular, R46 is also close to the iron binding sites corresponding to the catalytic center of ADO. Thus, activity enhancement by the mutations in region I may be due to perturbation of the active site residues.

Region II, including A17, P18, Q102, L161, R192, A196, E202, and V203, is located close to the substrate-entry/product-exit site of ADO [37] (Fig. 6). Therefore, mutations in this region may affect the open/close dynamics of the substrate-entry site, leading to enhanced activity. Consistent with this, previous studies have shown that the W178R mutation introduced in region II of 7942ADO enhanced ADO activity by removing the Trp residue that points towards the substrate and may interfere with substrate entry [39]. On the other hand, the increase in the amount of soluble ADO by the A17E and A196E mutations may be attributed to the stabilization of ADO that occurs by replacing exposed hydrophobic residues with hydrophilic residues.

Finally, region III, including D115, V121, Q165, R171, T174, V175, I179, R181, A184, and R239, is located close to the turns between α-helices that are far from the active site and the substrate binding site (Fig. 6). The I179L mutation that best improved activity is involved in this region. Similarly, E88, which is not included in either region, is located at a turn between the α-helices. Residues on loops between helices and/or strands have been shown to be important in determining the catalytic reactions of enzymes [67]. Hinge-like motions in the loop regions of enzymes have been suggested to facilitate large-scale, slower motions that produce catalytically competent states [68]. Thus, the introduction of mutations in region III may enhance activity and stability by modulating the structural dynamics of ADO, especially the open/close dynamics of the substrate-entry site near region III [69, 70]. In support of this, Shakeel et al. [70] recently reported that the A161E mutation in 73102ADO (corresponding to V173E in 7421ADO), which is introduced in region III, improves ADO activity at high temperatures [70].

In addition, it may be possible that mutations in ADO affect the interactions with AAR and/or the reducing system composed of Fd and FNR and then improve/reduce the activity and solubility of ADO. Future studies on the ADO residues that are essential for binding with AAR and Fd/FNR will clarify the reason for the mutational effects observed in the present study.

Taken together, the present results suggest that residues in regions I, II, and III are good targets for introducing mutations to improve ADO activity. In addition, stabilization of the ADO protein via removal of exposed hydrophobic residues may improve the solubility of ADO. A combination of these mutations will increase hydrocarbon production by ADO.

### Substrate specificity

Our previous studies showed that there is a difference in substrate specificity between AARs from marine and freshwater cyanobacteria, suggesting that the type of habitat of the host cyanobacteria may determine the substrate specificity of AAR [24]. In contrast, the present results indicate that the substrate specificity of ADO is not affected by the type of habitat of the host cyanobacteria (Fig. 2), suggesting that the residues essential for determining the substrate specificity of ADO are conserved among ADOs. This suggestion is supported by three lines of evidence. First, the substrate specificity of 7421ADO was not remarkably changed by any of the mutations at its non-conserved residues (Fig. 4). Second, the residues that constitute the substrate binding channel are reported to be conserved among ADOs from several cyanobacteria [38]. Third, mutations at conserved residues can change the substrate specificity of the ADO [33, 43]. Therefore, these results may suggest that both the activity and substrate specificity of ADO can be regulated separately by introducing mutations at non-conserved and conserved residues, respectively.

## Conclusions

In this study, we compared the hydrocarbon-producing activities of 10 representative ADOs and demonstrated that 7942ADO had the highest activity. By creating 37 mutants of the less active 7421ADO, in which a non-conserved residue was replaced by the corresponding residue in the highly active 7942ADO, we identified 20 non-conserved residues that are responsible for the high activity of ADO. Among them, the Q108K and R181E mutations increased both the activity and the amount of the soluble form of ADO, and eight mutations increased the ADO activity while maintaining more than 85% of the amount of soluble ADO. We also identified 13 other mutations that increased the amount of soluble ADO while maintaining more than 80% of the wild-type activity. Our results may be useful for generating combinatorial mutants of ADO that have both higher activity and higher amounts of the soluble protein in vivo, thereby producing higher yields of biohydrocarbons.

## Methods

### Plasmids

The DNA fragments encoding 6803ADO, 73102ADO, 7336ADO, 7421ADO, 7942ADO, 9443ADO, and *Te*ADO were prepared by overlap extension polymerase chain reaction. The codons were optimized for high expression in *E. coli* [71]. The codon-optimized DNA fragments coding for 9313ADO, 7425ADO, and *Pa*ADO were constructed by Eurofin Operon Biotechnologies (Tokyo, Japan). For the coexpression of AAR and ADO in *E. coli*, the pETDuet-1 coexpression vector (Merck Millipore, Darmstadt, Germany) was used, into which the codon-optimized DNA fragments of AAR and ADO were cloned via the *Nco*I and *Eco*RI restriction sites and the *Nde*I and *Avr*II restriction sites, respectively [22]. Both the AAR and ADO genes were preceded by a T7 promoter, *lac* operator, and ribosome binding site. 7942AAR was used for coexpression because it is known to have high aldehyde-producing activity [24]. All AAR and ADO proteins had a C-terminal extension of Gly-Ser-Ser-Gly and a 6 × His-tag. The ESPRESSO server, which estimates protein expression (http://cblab.meiyaku.jp/ESPRESSO/) [72], predicted that all the AAR and ADO constructs could be expressed in *E. coli*. The mutants of 7421ADO were constructed according to the protocols of the QuikChange site-directed mutagenesis kit (Agilent Technologies, Santa Clara, CA, USA).

### Hydrocarbon production in *E. coli* coexpressing AAR and ADO

Alkanes and alkenes were produced in *E. coli* coexpressing AAR and ADO, as previously described [22, 24]. Briefly, *E. coli* BL21(DE3)pLysS competent cells were transformed with the pETDuet-1 coexpression vector, containing both AAR and ADO genes, and inoculated onto a 2 × YT agarose plate containing carbenicillin (50 μg/mL) and chloramphenicol (34 μg/mL). The colonies on the plate were seeded into M9 medium containing ampicillin (50 μg/mL) and chloramphenicol (34 μg/mL) and pre-cultured at 37 °C overnight. The preculture was then seeded at an optical density of 0.1 at 600 nm into M9 medium containing 100 µM ammonium iron (II) sulfate and 1 mM isopropyl β-d-1-thiogalactoside. The culture was incubated in a 96 deep-well plate at 37 °C for 16 h. The cell culture was sonicated, and 500 µL of the cell lysate was centrifuged to separate the supernatant and pellet fractions, which were used to quantify the amounts of the soluble and insoluble forms of the AAR and ADO proteins by western blotting (for 10 representative ADOs) and SDS-PAGE (for the 7421ADO mutants). Next, 800 µL of the cell lysate was mixed with an equal amount of ethyl acetate by vortexing. The organic phase was separated from the aqueous phase by centrifugation and was subjected to GC–MS analysis using a Shimadzu gas chromatograph mass spectrometer GCMS-QP2010 Ultra (Shimadzu, Kyoto, Japan), as described elsewhere [22, 24].

The total amounts of hydrocarbons [pentadecane (C15:0), heptadecene (C17:1), and heptadecane (C17:0)] produced in the culture were normalized to the amount of the soluble form of the ADO protein expressed in *E. coli*, which was quantified using western blotting or SDS-PAGE. After normalization, the total amounts of hydrocarbons were compared with those of 73102ADO (for 10 representative ADOs) or the wild-type 7421ADO (for 7421ADO mutants), and the ratio was used as an index of hydrocarbon-producing activity of ADO. The measurements were carried out more than three times, and the means ± standard errors are shown.

### Western blotting

The amounts of the soluble and insoluble forms of ADO proteins were measured by SDS-PAGE or western blotting as previously described [22, 24]. Briefly, supernatant and pellet samples, prepared from the same amounts of cell lysates, were analyzed by SDS-PAGE using 12% gels. The proteins were electrotransferred onto a polyvinylidene difluoride membrane (Millipore), and the membrane was blocked with 100 mL of phosphate-buffered saline containing 5% skim milk. The membrane was incubated with an anti-His-tag antibody conjugated with horseradish peroxidase (MBL, Japan). The AAR and ADO proteins, both having a His-tag at the C-terminus, were visualized by color reactions with 5 mL of 3,3′,5,5′-tetramethylbenzidine solution (ATTO, Tokyo, Japan). Gel images of western blotting were acquired using a Gel Doc EZ imager (Bio-Rad, Hercules, CA, USA). Quantification of the bands of AAR and ADO was performed using ImageLab software (Bio-Rad).

### Analysis of amino acid sequences

A total of 134 orthologs of ADO were found on the BLAST server [73] using the amino acid sequence of 73102ADO as a query. The multiple sequence alignment of the 134 ADO amino acid sequences was carried out on the BLAST server. The phylogenetic tree of cyanobacteria based on the ADO amino acid sequences was drawn using NJplot [74]. The multiple sequence alignment of 10 representative ADOs was carried out using the Clustal X software [75].

The habitats of 134 cyanobacterial species were examined using MicrobeDB (http://microbedb.jp/), CyanoBase (http://genome.microbedb.jp/cyanobase/) [76], and the Pasteur Culture Collection of Cyanobacteria (PCC; http://cyanobacteria.web.pasteur.fr/). Ninety-three and 31 species are freshwater and marine cyanobacteria, respectively, and 10 species can live in both freshwater and marine environments.

## Additional files

**Additional file 1: Table S1.** Amino acid sequence identities (%) among the ADO sequences used in the present study.

**Additional file 2: Figure S1.** Phylogenetic tree of cyanobacterial ADOs based on ADO amino acid sequences. ADO sequence groups 1, 2, and 3 are shown in hatched *yellow*, *cyan*, and *magenta*, respectively. Host strains of ADOs used in the present study are indicated in *red*. *Vertical bars* show habitats of derived cyanobacteria: marine (*blue*), freshwater (*red*), and both marine and freshwater environments (*green*). The scale of the phylogenetic tree is shown in the *upper right corner.*

**Additional file 3: Figure S2.** GC–MS profiles of extracts from *E. coli* cell cultures coexpressing 7942AAR and ADO from one of 10 representative cyanobacteria. **a** Whole GC–MS profiles. Pentadecane (C15:0), heptadecene (C17:1), and heptadecane (C17:0) were eluted at retention times of 14.15, 16.32, and 16.54 min, respectively. The region in the black square is expanded and shown in Additional file 4: Figure S3c. **b** Peaks of pentadecane. **c** Peaks of heptadecene and heptadecane. Color codes for various ADOs are shown in panel (**a**).

**Additional file 4: Figure S3.** GC–MS profiles of fatty aldehydes and corresponding fatty alcohols. **a**, **b** GC–MS profiles of fatty aldehyde and alcohol standards, including hexadecanal (C16:0 aldehyde), hexadecanol (C16:0 alcohol), octadecanal (C18:0 aldehyde), and octadecanol (C18:0 alcohol), which were eluted at retention times of 17.69, 18.35, 19.74 and 20.32 min, respectively. The concentrations of the standards were 50 mg/L (**a**) and 10 mg/L (**b**). **c**, **d** GC–MS profiles of the extracts from *E. coli* cell culture expressing neither AAR nor ADO (**c**) and from *E. coli* coexpressing 7942AAR and 73102ADO (**d**). In panel (**d**), peaks 1, 2, and 3 correspond to hexadecanal, hexadecanol, and octadecenal, respectively, while these peaks are not present in panel (**c**).

**Additional file 5: Table S2.** The absolute amounts of hydrocarbons (in milligrams) produced in *E. coli* coexpressing ADO and 7942AAR per liter of *E. coli* cell culture using M9 medium.

**Additional file 6: Figure S4.** Solubility and protein expression levels of ADO and 7942AAR in *E. coli*. **a** Western blotting analysis of the supernatant and pellet fractions of the *E. coli* cell culture lysates. Images of triplicate experiments are shown. The *E. coli* cell culture coexpressing ADO and 7942AAR was sonicated and centrifuged to separate the supernatant (*S*) and pellet (*P*) fractions. *M* denotes the molecular weight markers. The bands for AAR (38.6 kDa) and ADO (26.5–28.3 kDa) are indicated by *arrows*. The results for *E. coli* transformed with the pETDuet-1 vector containing neither the AAR nor ADO gene are shown in *lanes* 17 and 18 as a control. In *lanes* 9 and 10, the 7425ADO protein migrated more slowly than the other ADOs (see text for details). **b** The amount of the soluble form of 7942AAR relative to that observed in *E. coli* coexpressing 73102ADO.

**Additional file 7: Table S3.** Charge and molecular weight of each ADO used in the present study.

**Additional file 8: Figure S5.** Correlation analysis among various properties of 10 representative ADOs. The relative amount of total hydrocarbon plotted against relative activity (**a**), relative amount of soluble ADO (**b**), solubility (**c**), and relative protein expression level of ADO (**d**). The relative activity of ADO plotted against the relative amount of soluble ADO (**e**), the relative protein expression level (**f**), and solubility (**g**). The relative amount of soluble ADO plotted against the relative protein expression level (**h**) and solubility (**i**). Solubility plotted against the relative protein expression level (**j**). In each panel, the *red continuous line* indicates a linear regression. The corresponding correlation coefficients, *r*, and the *p* values are shown.

**Additional file 9: Figure S6.** Correlation analysis of various properties of ADO with amino acid sequence identities. **a** The relative activity of ADO plotted against sequence identity (%) with the amino acid sequence of 7942ADO. **b**, **c** The solubility (**b**) and protein expression level (**c**) of ADO plotted against sequence identity (%) with the amino acid sequences of *Te*ADO and 7421ADO, respectively. **d** The amount of total hydrocarbon produced in *E. coli* coexpressing 7942AAR and ADO from one of 10 representative cyanobacteria plotted against sequence identity (%) with the amino acid sequence of *Te*ADO. **e** The relative amount of soluble ADO plotted against sequence identity (%) with the amino acid sequence of 7421ADO. In each panel, the *red continuous line* and *blue dotted line* respectively indicate a linear regression obtained using all data and that obtained without the data point for the highest value. The corresponding correlation coefficients, *r*, and the *p* values are shown.

**Additional file 10: Figure S7.** Correlation analysis among various properties of the 7421ADO mutants. **a**, **b** The relative amounts of hydrocarbons plotted against the solubility (**a**) and relative protein expression level of 7421ADO (**b**). **c** The relative activity of 7421ADO plotted against the relative protein expression level. **d** The solubility of ADO plotted against the relative protein expression level. In (**a**, **d**), the data point for R181E is highlighted. The *blue dotted line* indicates a linear regression obtained without using the data for R181E.

## Abbreviations

6803ADO: ADO from *Synechocystis* sp. PCC 6803
73102ADO: ADO from *Nostoc punctiforme* PCC 73102
7336ADO: ADO from *Synechococcus* sp. PCC 7336
7421ADO: ADO from *Gloeobacter violaceus* PCC 7421
7425ADO: ADO from *Cyanothece* sp. PCC 7425
7942AAR: AAR from *Synechococcus elongatus* PCC 7942
7942ADO: ADO from *Synechococcus elongatus* PCC 7942
9313ADO: ADO from *Prochlorococcus marinus* MIT 9313
9443ADO: ADO from *Microcystis aeruginosa* PCC 9443
AAR: acyl-ACP reductase
ACP: acyl carrier protein
ADO: aldehyde-deformylating oxygenase
CoA: coenzyme A
Fd: ferredoxin
FNR: ferredoxin NADP^+^ reductase
*Pa*ADO: ADO from *Planktothrix agardhii* NIVA-CYA 126/8
GC-MS: gas chromatography-mass spectrometry
SDS-PAGE: sodium dodecyl sulfate–polyacrylamide gel electrophoresis
*Te*ADO: ADO from *Thermosynechococcus elongatus* BP-1
WT: wild type
