# Supplementary material for: Identification of non-conserved residues essential for improving the hydrocarbon-producing activity of cyanobacterial aldehyde-deformylating oxygenase
Source: Biotechnol Biofuels. 2019 Apr 17;12:89. doi: 10.1186/s13068-019-1409-8 (PMC6469105; doi:10.1186/s13068-019-1409-8)
Supplement: Supplementary file 1 — Additional file 1: Table S1. Amino acid sequence identities (%) among the ADO sequences used in the present study. [file 13068_2019_1409_MOESM1_ESM.pdf]

Table S1. Amino acid sequence identities (%) among the ADO sequences used in the present study.

	Group	7942 ADO	6803 ADO	9313 ADO	73102 ADO	*Pa* ADO	7425 ADO	9443 ADO	*Te* ADO	7421 ADO	7336 ADO
7942ADO	1	–	72	60	73	77	71	75	70	63	65
6803ADO	1	72	–	68	75	73	73	80	74	65	63
9313ADO	2	60	68	–	62	64	66	65	65	57	57
73102ADO	1	73	75	62	–	78	72	79	76	63	67
*Pa*ADO	1	77	73	64	78	–	75	75	70	64	64
7425ADO	1	71	73	66	72	75	–	73	73	66	65
9443ADO	1	75	80	65	79	75	73	–	73	65	64
*Te*ADO	1	70	74	65	76	70	73	73	–	64	62
7421ADO	3	63	65	57	63	64	66	65	64	–	66
7336ADO	3	65	63	57	67	64	65	64	62	66	–
