# Supplementary figures and images for: Identification of non-conserved residues essential for improving the hydrocarbon-producing activity of cyanobacterial aldehyde-deformylating oxygenase
Source: Biotechnol Biofuels. 2019 Apr 17;12:89. doi: 10.1186/s13068-019-1409-8 (PMC6469105; doi:10.1186/s13068-019-1409-8)

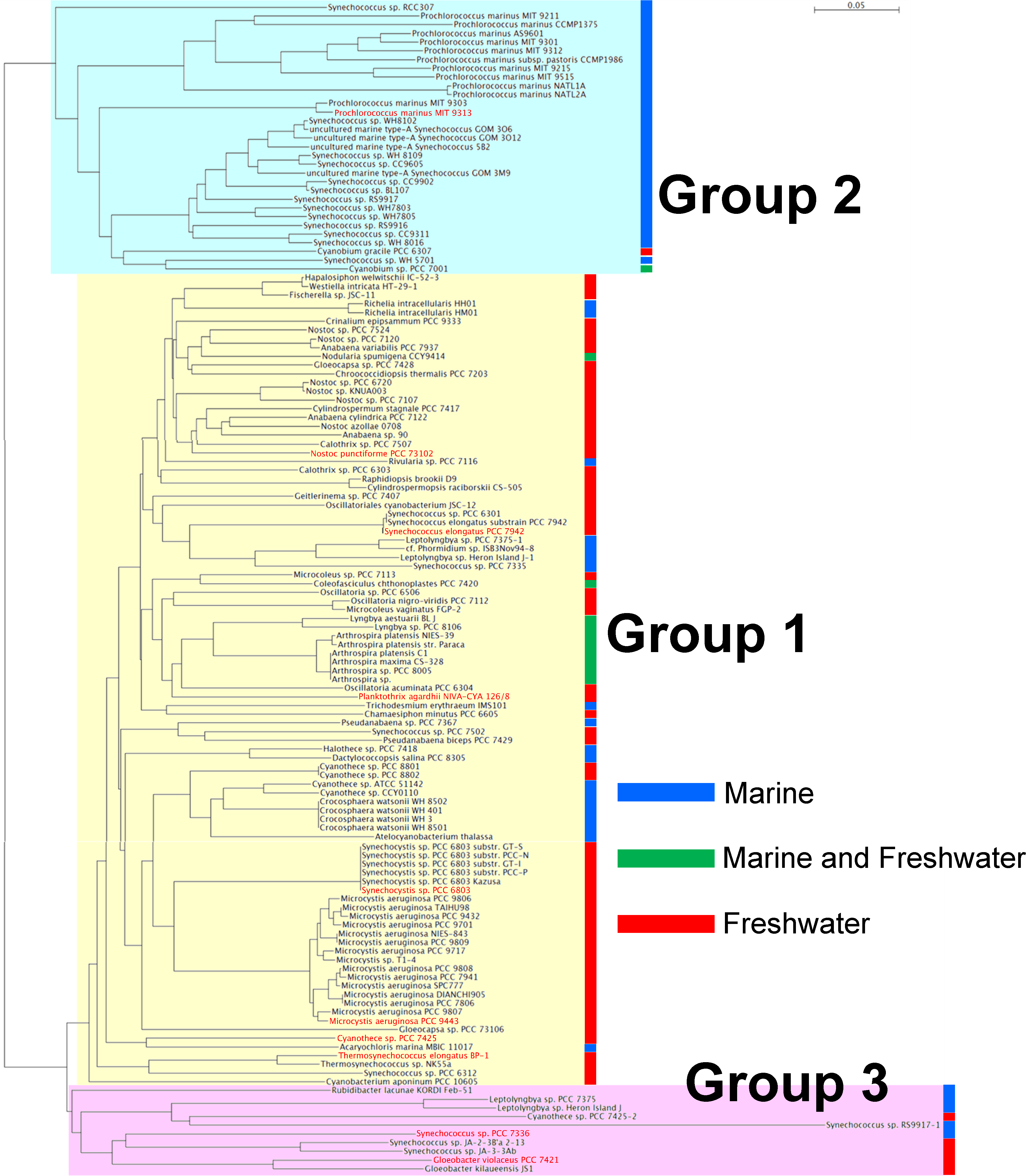

Supplement: Supplementary file 2 — Additional file 2: Figure S1. Phylogenetic tree of cyanobacterial ADOs based on ADO amino acid sequences. ADO sequence groups 1, 2, and 3 are shown in hatched yellow, cyan, and magenta, respectively. Host strains of ADOs used in the present study are indicated in red. Vertical bars show habitats of derived cyanobacteria: marine (blue), freshwater (red), and both marine and freshwater environments (green). The scale of the phylogenetic tree is shown in the upper right corner. [file 13068_2019_1409_MOESM2_ESM.tif]

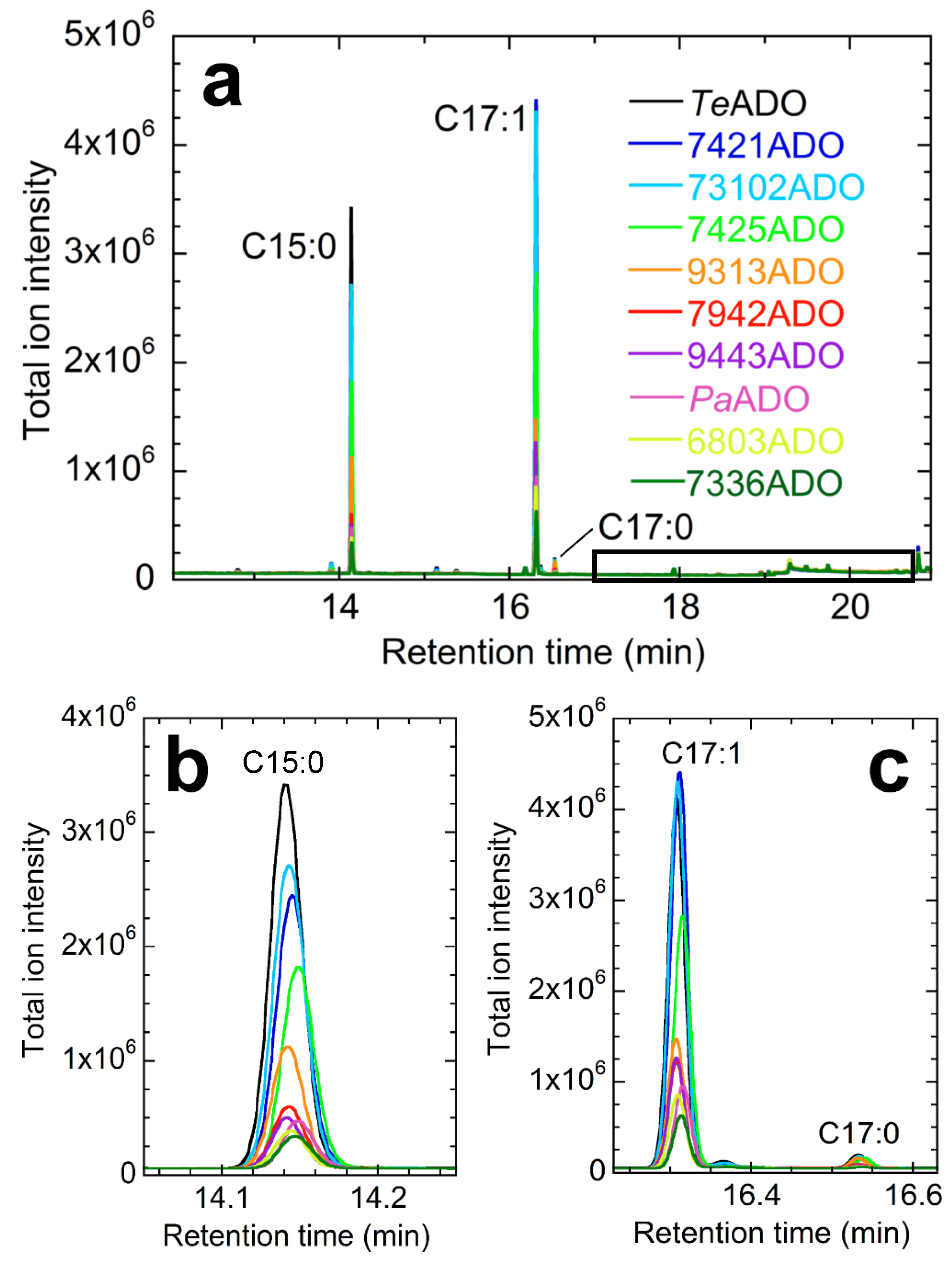

Supplement: Supplementary file 3 — Additional file 3: Figure S2. GC–MS profiles of extracts from E. coli cell cultures coexpressing 7942AAR and ADO from one of 10 representative cyanobacteria. a Whole GC–MS profiles. Pentadecane (C15:0), heptadecene (C17:1), and heptadecane (C17:0) were eluted at retention times of 14.15, 16.32, and 16.54 min, respectively. The region in the black square is expanded and shown in Additional file 4: Figure S3c. b Peaks of pentadecane. c Peaks of heptadecene and heptadecane. Color codes for various ADOs are shown in panel (a). [file 13068_2019_1409_MOESM3_ESM.tif]

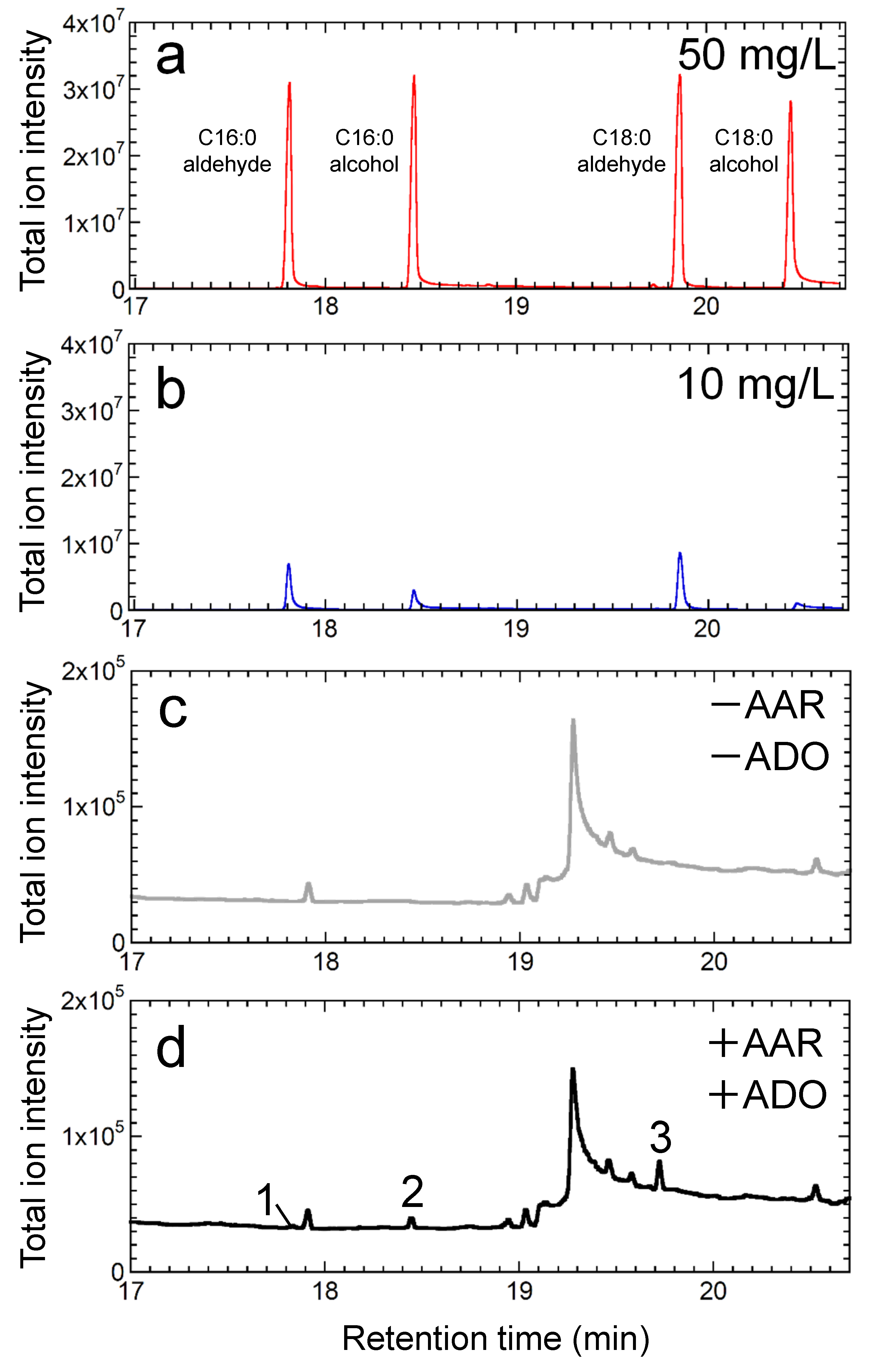

Supplement: Supplementary file 4 — Additional file 4: Figure S3. GC–MS profiles of fatty aldehydes and corresponding fatty alcohols. a, b GC–MS profiles of fatty aldehyde and alcohol standards, including hexadecanal (C16:0 aldehyde), hexadecanol (C16:0 alcohol), octadecanal (C18:0 aldehyde), and octadecanol (C18:0 alcohol), which were eluted at retention times of 17.69, 18.35, 19.74 and 20.32 min, respectively. The concentrations of the standards were 50 mg/L (a) and 10 mg/L (b). c, d GC–MS profiles of the extracts from E. coli cell culture expressing neither AAR nor ADO (c) and from E. coli coexpressing 7942AAR and 73102ADO (d). In panel (d), peaks 1, 2, and 3 correspond to hexadecanal, hexadecanol, and octadecenal, respectively, while these peaks are not present in panel (c). [file 13068_2019_1409_MOESM4_ESM.tif]

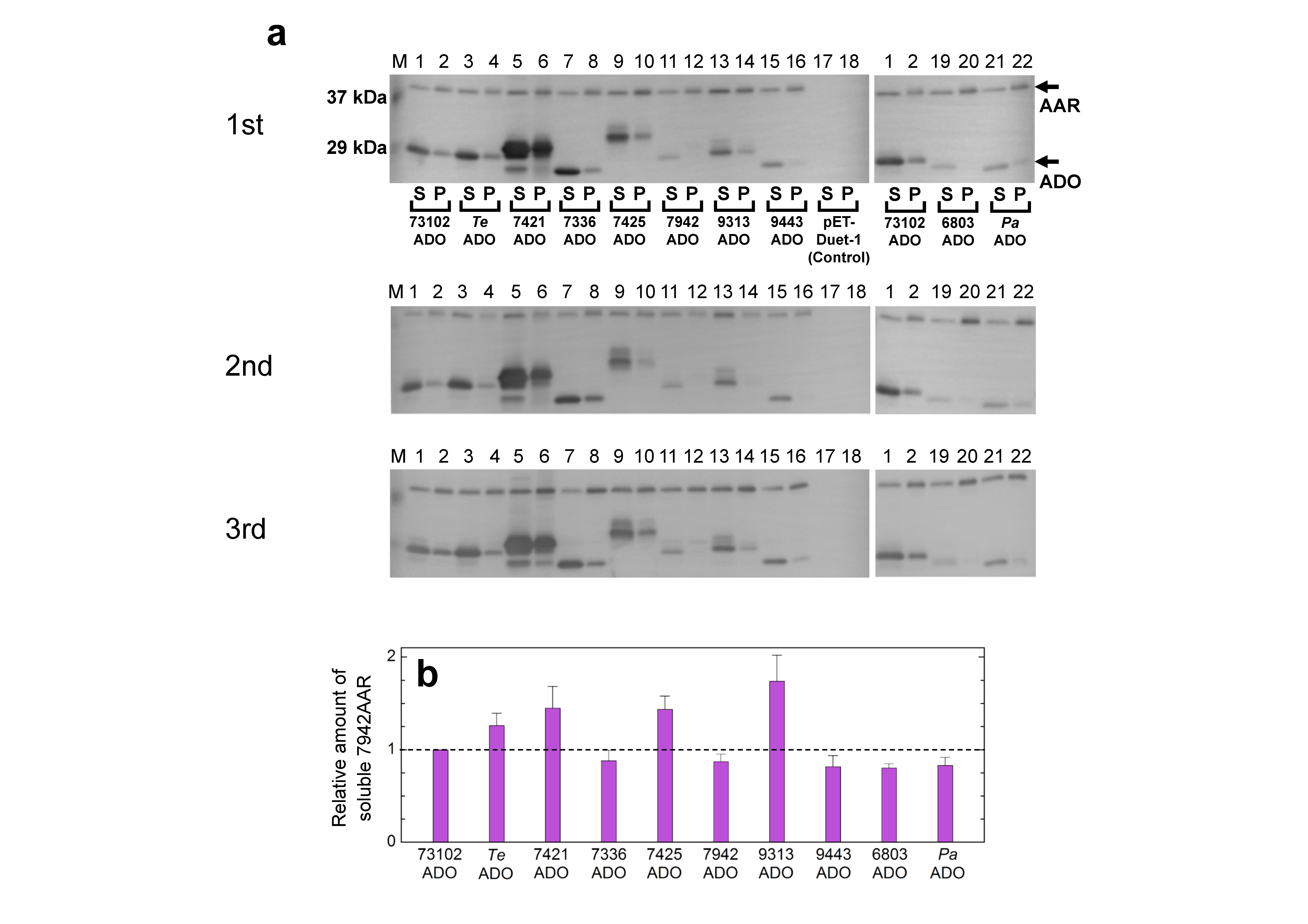

Supplement: Supplementary file 6 — Additional file 6: Figure S4. Solubility and protein expression levels of ADO and 7942AAR in E. coli. a Western blotting analysis of the supernatant and pellet fractions of the E. coli cell culture lysates. Images of triplicate experiments are shown. The E. coli cell culture coexpressing ADO and 7942AAR was sonicated and centrifuged to separate the supernatant (S) and pellet (P) fractions. M denotes the molecular weight markers. The bands for AAR (38.6 kDa) and ADO (26.5–28.3 kDa) are indicated by arrows. The results for E. coli transformed with the pETDuet-1 vector containing neither the AAR nor ADO gene are shown in lanes 17 and 18 as a control. In lanes 9 and 10, the 7425ADO protein migrated more slowly than the other ADOs (see text for details). b The amount of the soluble form of 7942AAR relative to that observed in E. coli coexpressing 73102ADO. [file 13068_2019_1409_MOESM6_ESM.tif]

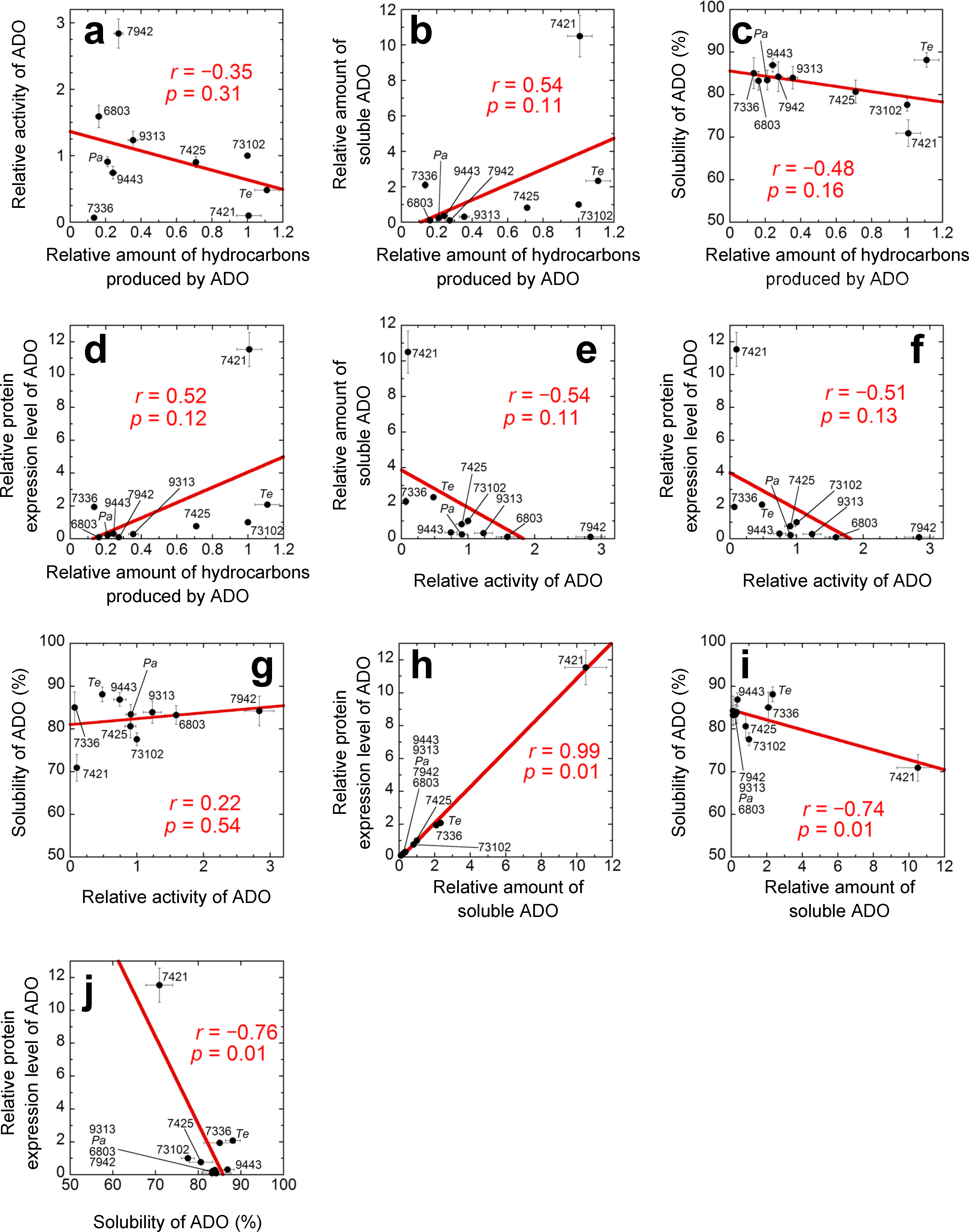

Supplement: Supplementary file 8 — Additional file 8: Figure S5. Correlation analysis among various properties of 10 representative ADOs. The relative amount of total hydrocarbon plotted against relative activity (a), relative amount of soluble ADO (b), solubility (c), and relative protein expression level of ADO (d). The relative activity of ADO plotted against the relative amount of soluble ADO (e), the relative protein expression level (f), and solubility (g). The relative amount of soluble ADO plotted against the relative protein expression level (h) and solubility (i). Solubility plotted against the relative protein expression level (j). In each panel, the red continuous line indicates a linear regression. The corresponding correlation coefficients, r, and the p values are shown. [file 13068_2019_1409_MOESM8_ESM.tif]

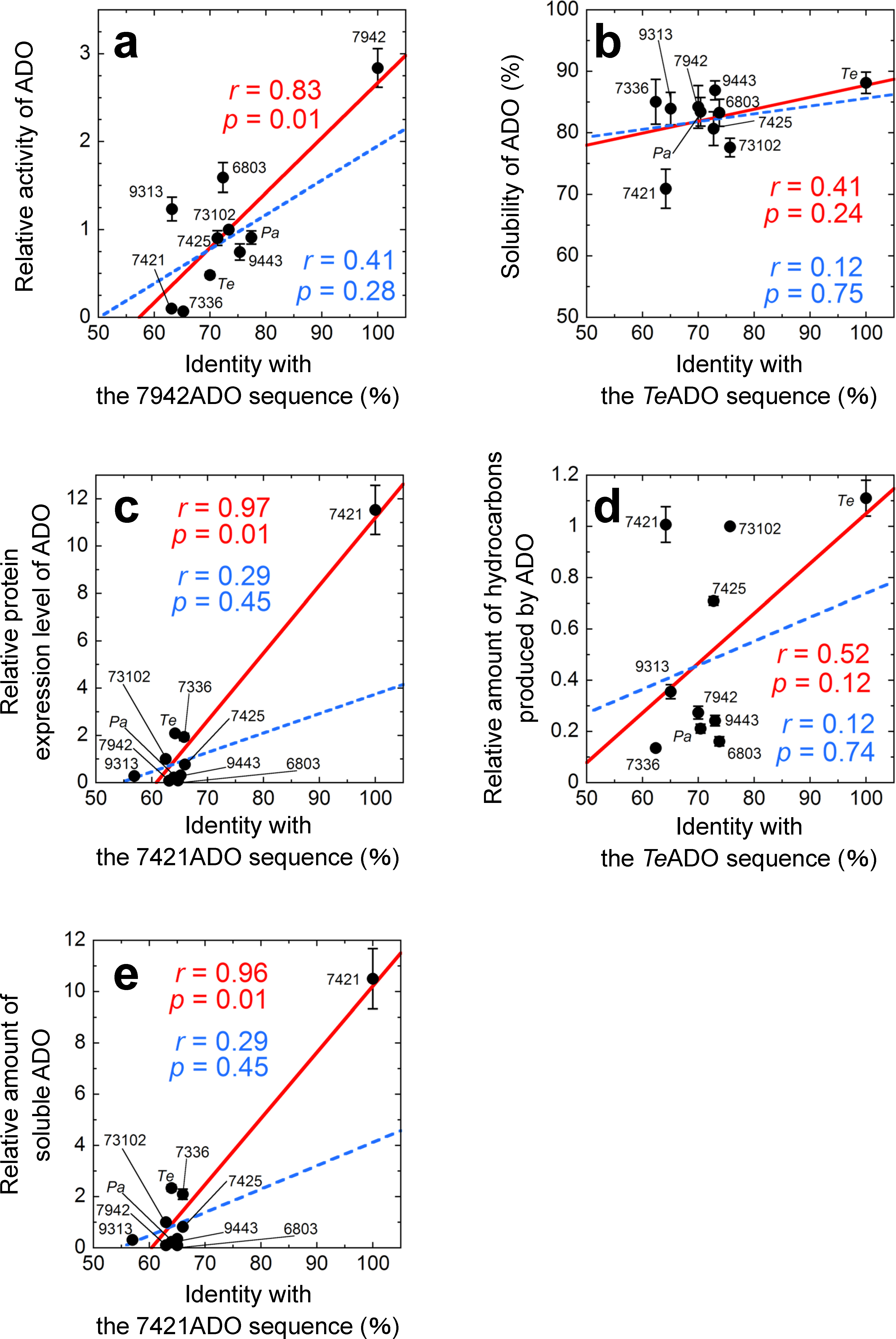

Supplement: Supplementary file 9 — Additional file 9: Figure S6. Correlation analysis of various properties of ADO with amino acid sequence identities. a The relative activity of ADO plotted against sequence identity (%) with the amino acid sequence of 7942ADO. b, c The solubility (b) and protein expression level (c) of ADO plotted against sequence identity (%) with the amino acid sequences of TeADO and 7421ADO, respectively. d The amount of total hydrocarbon produced in E. coli coexpressing 7942AAR and ADO from one of 10 representative cyanobacteria plotted against sequence identity (%) with the amino acid sequence of TeADO. e The relative amount of soluble ADO plotted against sequence identity (%) with the amino acid sequence of 7421ADO. In each panel, the red continuous line and blue dotted line respectively indicate a linear regression obtained using all data and that obtained without the data point for the highest value. The corresponding correlation coefficients, r, and the p values are shown. [file 13068_2019_1409_MOESM9_ESM.tif]

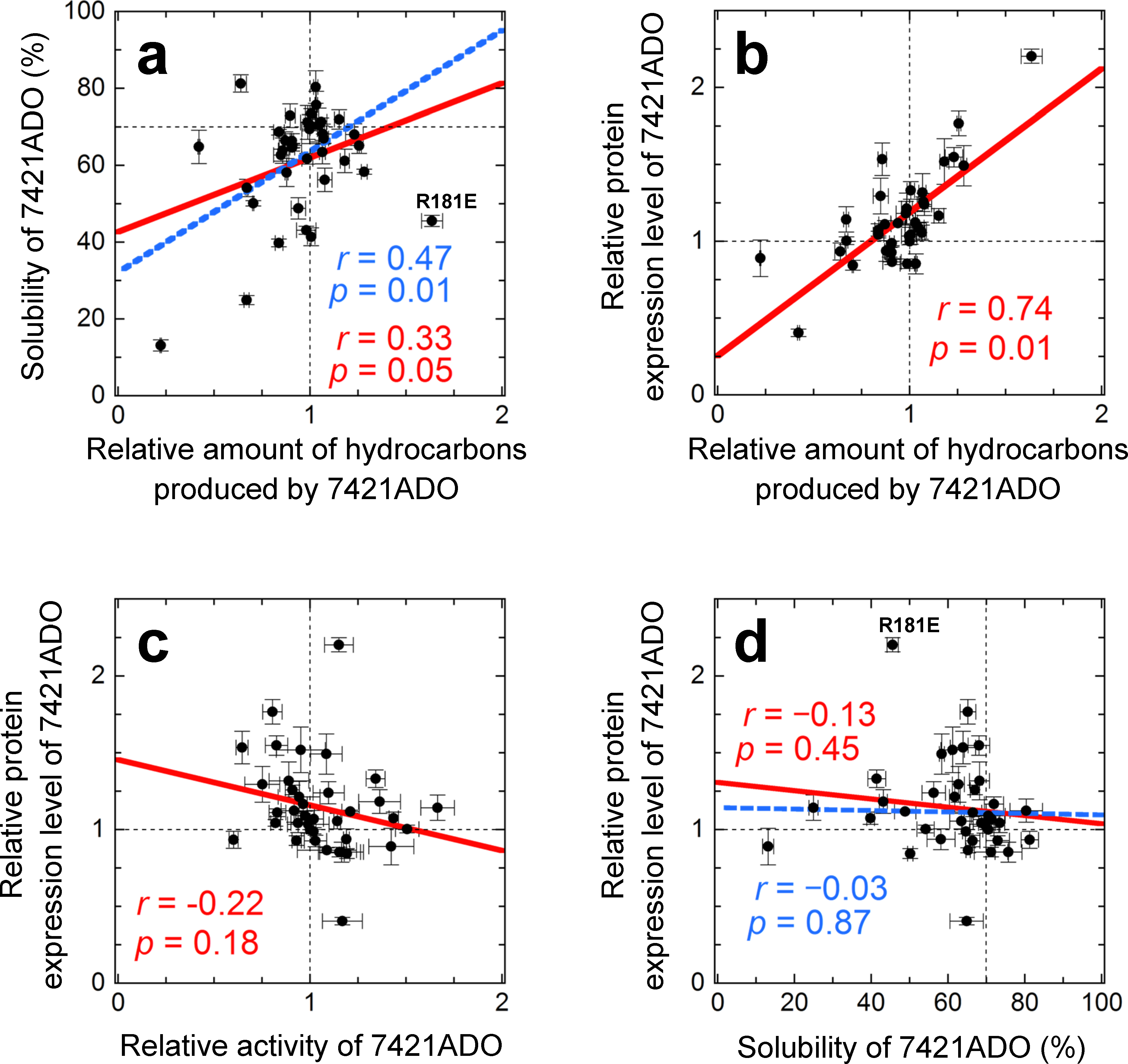

Supplement: Supplementary file 10 — Additional file 10: Figure S7. Correlation analysis among various properties of the 7421ADO mutants. a, b The relative amounts of hydrocarbons plotted against the solubility (a) and relative protein expression level of 7421ADO (b). c The relative activity of 7421ADO plotted against the relative protein expression level. d The solubility of ADO plotted against the relative protein expression level. In (a, d), the data point for R181E is highlighted. The blue dotted line indicates a linear regression obtained without using the data for R181E. [file 13068_2019_1409_MOESM10_ESM.tif]
