# Supplementary material for: Identification of non-conserved residues essential for improving the hydrocarbon-producing activity of cyanobacterial aldehyde-deformylating oxygenase
Source: Biotechnol Biofuels. 2019 Apr 17;12:89. doi: 10.1186/s13068-019-1409-8 (PMC6469105; doi:10.1186/s13068-019-1409-8)
Supplement: Supplementary file 5 — Additional file 5: Table S2. The absolute amounts of hydrocarbons (in milligrams) produced in E. coli coexpressing ADO and 7942AAR per liter of E. coli cell culture using M9 medium. [file 13068_2019_1409_MOESM5_ESM.pdf]

Table S2. The absolute amounts of hydrocarbons (in milligrams) produced in *E. coli* coexpressing ADO and 7942AAR per liter of *E. coli* cell culture using the M9 medium.

	Total (mg/L)	Pentadecane (C15:0) (mg/L)	Heptadecene (C17:1) (mg/L)	Heptadecane (C17:0) (mg/L)
7942ADO	3.3 ± 0.3	1.00 ± 0.09	2.20 ± 0.21	0.09 ± 0.01
6803ADO	1.9 ± 0.2	0.53 ± 0.06	1.37 ± 0.15	0.04 ± 0.01
9313ADO	4.3 ± 0.3	1.74 ± 0.13	2.33 ± 0.19	0.18 ± 0.01
73102ADO	12.0 ± 0.1	4.72 ± 0.03	7.15 ± 0.04	0.21 ± 0.01
*Pa*ADO	2.5 ± 0.2	0.78 ± 0.07	1.70 ± 0.15	0.05 ± 0.01
7425ADO	8.5 ± 0.2	3.32 ± 0.07	4.99 ± 0.14	0.21 ± 0.01
9443ADO	2.9 ± 0.2	0.77 ± 0.06	2.08 ± 0.19	0.05 ± 0.01
*Te*ADO	13.3 ± 0.8	5.90 ± 0.37	7.18 ± 0.46	0.24 ± 0.01
7421ADO	12.1 ± 0.8	4.16 ± 0.29	7.75 ± 0.53	0.18 ± 0.01
7336ADO	1.6 ± 0.1	0.49 ± 0.04	1.10 ± 0.09	0.03 ± 0.01
