# Supplementary material for: Identification of non-conserved residues essential for improving the hydrocarbon-producing activity of cyanobacterial aldehyde-deformylating oxygenase
Source: Biotechnol Biofuels. 2019 Apr 17;12:89. doi: 10.1186/s13068-019-1409-8 (PMC6469105; doi:10.1186/s13068-019-1409-8)
Supplement: Supplementary file 7 — Additional file 7: Table S3. Charge and molecular weight of each ADO used in the present study. [file 13068_2019_1409_MOESM7_ESM.pdf]

Table S3. Charge and molecular weight of ADOs used in the present study.

	Charge at pH 8.8	Molecular weight (kDa)
*Te*ADO	–12.6	27.3
7421ADO	–14.8	27.5
73102ADO	–18.1	27.4
9443ADO	–18.5	27.4
6803ADO	–18.7	27.3
*Pa*ADO	–19.0	27.2
9313ADO	–19.9	28.3
7942ADO	–19.9	27.5
7336ADO	–20.9	26.5
7425ADO	–21.7	27.5

Charge at pH 8.8 and molecular weight of ADOs with a C-terminal His-tag were calculated by the Protein Calculator v3.4 (http://protcalc.sourceforge.net/).
